# Intersecting Pathways: The Role of Metabolic Dysregulation, Gastrointestinal Microbiome, and Inflammation in Acute Ischemic Stroke Pathogenesis and Outcomes

**DOI:** 10.3390/jcm13144258

**Published:** 2024-07-21

**Authors:** Jarosław Nuszkiewicz, Beata Kukulska-Pawluczuk, Katarzyna Piec, Dorian Julian Jarek, Karina Motolko, Karolina Szewczyk-Golec, Alina Woźniak

**Affiliations:** 1 Department of Medical Biology and Biochemistry, Faculty of Medicine, Ludwik Rydygier Collegium Medicum in Bydgoszcz, Nicolaus Copernicus University in Toruń, 24 Karłowicza St., 85-092 Bydgoszcz, Poland; karosz@cm.umk.pl; 2 Department of Neurology, Faculty of Medicine, Ludwik Rydygier Collegium Medicum in Bydgoszcz, Nicolaus Copernicus University in Toruń, 9 M. Skłodowskiej—Curie St., 85-094 Bydgoszcz, Poland; bkukulska@cm.umk.pl (B.K.-P.); katarzyna.piec@cm.umk.pl (K.P.); 3 Student Research Club of Medical Biology and Biochemistry, Department of Medical Biology and Biochemistry, Faculty of Medicine, Ludwik Rydygier Collegium Medicum in Bydgoszcz, Nicolaus Copernicus University in Toruń, 24 Karłowicza St., 85-092 Bydgoszcz, Poland; 309897@stud.umk.pl; 4 Student Research Club of Neurology, Department of Neurology, Faculty of Medicine, Ludwik Rydygier Collegium Medicum in Bydgoszcz, Nicolaus Copernicus University in Toruń, 9 M. Skłodowskiej—Curie St., 85-094 Bydgoszcz, Poland; 300910@stud.umk.pl

**Keywords:** acute ischemic stroke, adipokines, biomarkers, brain-gut axis, gastrointestinal microbiome, inflammation, lipopolysaccharides, metabolic dysregulation, neuroinflammation, obesity

## Abstract

Acute ischemic stroke (AIS) remains a major cause of mortality and long-term disability worldwide, driven by complex and multifaceted etiological factors. Metabolic dysregulation, gastrointestinal microbiome alterations, and systemic inflammation are emerging as significant contributors to AIS pathogenesis. This review addresses the critical need to understand how these factors interact to influence AIS risk and outcomes. We aim to elucidate the roles of dysregulated adipokines in obesity, the impact of gut microbiota disruptions, and the neuroinflammatory cascade initiated by lipopolysaccharides (LPS) in AIS. Dysregulated adipokines in obesity exacerbate inflammatory responses, increasing AIS risk and severity. Disruptions in the gut microbiota and subsequent LPS-induced neuroinflammation further link systemic inflammation to AIS. Advances in neuroimaging and biomarker development have improved diagnostic precision. Here, we highlight the need for a multifaceted approach to AIS management, integrating metabolic, microbiota, and inflammatory insights. Potential therapeutic strategies targeting these pathways could significantly improve AIS prevention and treatment. Future research should focus on further elucidating these pathways and developing targeted interventions to mitigate the impacts of metabolic dysregulation, microbiome imbalances, and inflammation on AIS.

## 1. Introduction

Acute ischemic stroke (AIS) remains one of the most significant challenges in global health, standing as a leading cause of mortality and long-term disability worldwide [1]. Despite significant advances in acute management and preventive strategies, the incidence of AIS continues to rise, fueled by aging populations and the increasing prevalence of lifestyle-related risk factors such as obesity, diabetes, and hypertension [2]. These traditional risk factors, while critically important, do not fully account for the variability in AIS occurrence and outcomes. This gap has led researchers to investigate less-understood mechanisms involving metabolic dysregulation, gut microbiota, and systemic inflammation. Inflammation is the tissue’s response to harmful stimuli like pathogens or damaged cells, involving immune cells, blood vessels, and molecular mediators to eliminate injury causes and promote repair [3]. However, chronic inflammation can contribute to diseases such as cardiovascular and neurodegenerative conditions [3]. This suggests that additional, less-understood mechanisms are influencing the pathogenesis and progression of cerebral ischemia (CI).

Emerging research has begun to uncover the intricate relationships between metabolic dysregulation, the gut microbiota, and systemic inflammation, highlighting their potential to significantly impact AIS risk and recovery [4,5]. Metabolic dysregulation refers to the abnormal functioning of metabolic processes in the body, which can lead to conditions such as obesity, insulin resistance, and dyslipidemia [6]. Metabolic dysregulation has been robustly associated with an increased risk of cardiovascular diseases (CVDs), including AIS [7]. This association is mediated in part by adipokines—bioactive cytokines secreted by adipose tissue—which exert complex effects on systemic inflammation and endothelial function [8]. Adipokines play a crucial role in vascular health and metabolic homeostasis, but, in the context of obesity, their dysregulated production contributes to a pro-inflammatory state that may precipitate vascular pathologies leading to AIS [9].

Moreover, the gut microbiota, an often overlooked contributor to health and disease, consists of trillions of microorganisms, including bacteria, viruses, fungi, and protozoa, residing in the gastrointestinal tract [10]. These microorganisms play a crucial role in digestion, immune function, and overall health by maintaining a balanced internal environment [10]. The gut microbiota interacts extensively with the host’s metabolism and immune system, playing a pivotal role in maintaining metabolic balance and modulating inflammatory pathways [10]. The gut-brain axis, a complex communication network involving immune, endocrine, and neural mediators, connects alterations in the gut microbiota to neuroinflammatory processes that may play a significant role in AIS pathology [11,12]. Notably, the translocation of gut-derived lipopolysaccharides (LPS) into the systemic circulation—a condition often exacerbated by gut dysbiosis and increased intestinal permeability—can trigger widespread inflammatory responses [13]. These responses not only affect the gastrointestinal tract but also have profound impacts on systemic and particularly cerebrovascular health [13].

The systemic inflammatory state induced by metabolic dysregulation and changes in gut microbiota actively participates in AIS pathogenesis [14]. Chronic and acute inflammation mediates atherogenic processes, as evidenced by the secretion of various inflammatory mediators by adipose tissue in obese individuals, compounding traditional AIS risk factors [14]. Inflammation, both chronic and acute, mediates atherogenic processes that predispose individuals to ischemia [15]. This is evidenced by the active secretion of a variety of inflammatory mediators by the adipose tissue in obese individuals, which compounds the effects of traditional AIS risk factors like hypertension and hyperlipidemia [16].

The interplay between peripheral inflammation and central neuroinflammatory processes, mediated by cytokines and immune cells traversing a compromised blood-brain barrier (BBB), highlights the potential of systemic interventions to not only mitigate AIS risk but also modify its outcomes [17].

In the context of AIS, the transition from systemic inflammation to neuroinflammation involves several neural substrates and mechanisms. Key brain regions affected include the hippocampus, cortex, and basal ganglia, which are highly susceptible to inflammatory damage [18,19]. Specific cell types, such as microglia and astrocytes, play crucial roles [20]. Microglia, the resident immune cells of the brain, become activated in response to systemic inflammatory signals, releasing pro-inflammatory cytokines that exacerbate neuronal injury [20]. Similarly, astrocytes, which support neuronal function and maintain the BBB, undergo reactive astrogliosis, contributing to both protective and detrimental outcomes [20]. The disruption of the BBB, a hallmark of AIS, allows peripheral immune cells and inflammatory mediators to infiltrate the brain, further propagating neuroinflammation [21]. Understanding these substrates and mechanisms is essential for developing interventions that can mitigate the neuroinflammatory responses in AIS.

Despite the progress made in understanding the pathophysiology of AIS, significant gaps remain in translating these findings into clinical practice. A comprehensive integration of how metabolic dysregulation, gut microbiota alterations, and systemic inflammation jointly contribute to AIS is lacking. Another critical gap in current understanding is the lack of longitudinal studies examining the dynamic interactions between metabolic health, gut microbiota composition, and systemic inflammation over time. Understanding how these factors evolve and influence each other in the long term could provide deeper insights into preventive measures and the timing of interventions for AIS. Addressing these gaps could lead to the development of targeted strategies that not only mitigate the risk but also improve the prognosis of AIS patients. Furthermore, research is needed to determine which inflammatory factors or models determine the risk of specific etiopathogenetic factors of stroke, such as large artery atherosclerosis (LAA), cardioembolism (CE), and cerebral small vessel disease (CSVD). Additionally, understanding whether environmental, behavioral, or genetic factors have a greater impact on the course of generalized inflammation, which underlies stroke etiopathogenesis, is crucial. Finally, identifying the optimal timing and targets for inhibiting the inflammatory cascade to prevent metabolic, organ, and functional dysfunction is essential for improving AIS outcomes. With numerous cause-and-effect connections, therapies targeting single pathways may prove suboptimal for protecting patients from developing risk factors for vascular diseases.

This narrative review explores the intricate relationships between systemic inflammation, gut microbiota alterations, and metabolic dysfunctions such as obesity and their collective impact on AIS. With AIS ranking as a leading cause of mortality and long-term disability worldwide, understanding its complex mechanisms is more critical than ever. The review aims to synthesize current research to elucidate how these interconnected pathways influence the risk, severity, and recovery from CI. It explores the mechanisms by which external factors like pathogens and their components, such as LPS, initiate inflammatory responses that contribute to AIS pathogenesis. By integrating insights from metabolic health, microbiota research, and inflammation, the article seeks to outline new diagnostic tools, including emerging biomarkers and neuroimaging techniques, and to highlight potential therapeutic strategies that could lead to more effective prevention and treatment of AIS. This comprehensive review aims to enhance clinical outcomes by providing a holistic view of the metabolic, microbial, and inflammatory factors in AIS, pushing forward the boundaries of current AIS research and clinical practice.

## 2. Inflammation in AIS: Basic Mechanisms and External Factors

### 2.1. Cellular and Molecular Foundations of Inflammation

Inflammation is a fundamental physiological response by the immune system designed to protect the body from injury and infection [22]. It serves as both a defense mechanism and a healing process, triggered when tissues are damaged by bacteria, trauma, toxins, heat, or any other cause [22]. The main goal of inflammation is to eliminate the cause of cell injury, clear out necrotic cells, and initiate tissue repair [23]. Inflammation is classified into acute and chronic types [22]. Acute inflammation is short-lived, characterized by edema, redness, heat, and pain, resolving upon the removal of the offending agent [24]. Key events include increased blood flow, vascular permeability, and leukocyte migration, primarily neutrophils, to the injury site [25]. Chronic inflammation can persist for months or years due to persistent infections, prolonged exposure to toxic agents, or autoimmune diseases, often leading to tissue destruction and scarring [26]. In addition to localized inflammation within the central nervous system (CNS), systemic inflammation often plays a critical role in cerebral outcomes following AIS [27]. Factors such as underlying health conditions, systemic infections, or chronic inflammatory diseases can exacerbate or modulate inflammatory responses within the brain, thereby influencing the severity and progression of CI [28]. Understanding the interaction between systemic and local inflammation is crucial for developing comprehensive therapeutic strategies that address both dimensions of inflammatory response.

Cytokines are versatile signaling proteins that orchestrate the immune response to infection and injury by modulating the behavior of cells both within and outside the immune system [29]. Although cytokines are commonly categorized as pro-inflammatory and anti-inflammatory based on their roles in promoting or suppressing inflammation, this dichotomy oversimplifies their complex nature and context-dependent actions [30]. Pro-inflammatory cytokines generally initiate and sustain inflammatory responses, mobilizing and activating immune cells to defend against pathogens or heal injured tissues [31]. However, when produced in excess, pro-inflammatory cytokines can lead to tissue damage and are implicated in various chronic inflammatory conditions, including the neuroinflammation observed in AIS [31]. On the other hand, anti-inflammatory cytokines help to dampen inflammatory responses and facilitate tissue recovery, ensuring that inflammation ceases once a threat is neutralized to prevent excessive tissue damage [32]. Beyond this binary classification, cytokines can also be categorized based on their functional roles in different immune pathways. Some cytokines, such as those involved in the acute phase response, primarily act to mobilize immediate defenses and coordinate systemic responses, including fever and the production of other cytokines and acute phase proteins [33]. This nuanced view acknowledges that the function of cytokines can vary significantly depending on the tissue context, the type and phase of the immune response, and the presence of other cytokines. For instance, certain cytokines may have pro-inflammatory effects in the context of acute infection but support tissue regeneration and healing in other circumstances [34]. This functional plasticity makes cytokines both fascinating and challenging targets for therapeutic intervention, particularly in conditions like AIS, where the role of inflammation is both protective and pathological. In targeting cytokine pathways for therapeutic purposes, especially in complex diseases such as AIS, it is essential to consider not only the balance between pro-inflammatory and anti-inflammatory cytokines but also how interventions might affect other immune functions. Modulating cytokine activity offers a promising avenue for mitigating the detrimental aspects of inflammation while preserving or enhancing its beneficial effects, aiming for an optimal recovery following CI.

In the CNS, resident immune cells such as microglia and astrocytes play critical roles in maintaining homeostasis and responding to injury [35]. Microglia, the primary immune cells of the CNS, play a crucial role both in health and disease. In a healthy brain, they continuously monitor the environment, eliminating debris and dead cells and supporting neuronal function by releasing growth factors [36]. This surveillance is critical for maintaining the CNS’s integrity. However, upon injury or in disease states such as AIS, microglia quickly become activated, transforming from a ramified to an amoeboid shape [37]. This change enables them to isolate damaged areas, clear cellular debris, and release pro-inflammatory cytokines that attract additional immune cells to the injury site [38]. The activation of microglia can have dual outcomes—neurotoxicity and neuroprotection—depending on the type of activation. There are two major activation states: classical (M1 type) and alternative (M2 type) [39]. M1 microglia secrete pro-inflammatory cytokines and neurotoxic mediators that exacerbate neuronal damage, whereas M2 microglia promote a reparative, anti-inflammatory response [40,41]. The balance between these two activation states determines the fate of damaged neurons, highlighting the complexity of microglial function in response to CNS injury. Overactivation of microglia, particularly of the M1 type, can worsen injury by releasing excessive neurotoxic substances, including reactive oxygen species (ROS) and pro-inflammatory cytokines, thus contributing to neuronal death [42]. Fine regulation of M1/M2 microglial activation is therefore crucial, offering significant therapeutic value in minimizing damage and maximizing protection [43]. Astrocytes, another pivotal glial cell type, are involved in numerous functions, including neurotransmitter uptake and recycling, energy metabolism, and the maintenance of the BBB [44]. Under pathological conditions, astrocytes undergo proliferation and hypertrophy in a process known as reactive astrogliosis [45]. Reactive astrocytes can form a glial scar, which acts to protect surrounding healthy neurons by compartmentalizing the damaged area but can also inhibit axonal regeneration and functional recovery [46]. Similar to microglia, astrocytes can release both neuroprotective factors such as neurotrophins, and pro-inflammatory mediators, influencing the balance between neuroprotection and neurotoxicity [47]. The crosstalk between microglia, astrocytes, and endothelial cells is pivotal in regulating the integrity of the BBB and mediating the infiltration of peripheral immune cells [48]. Activated microglia can induce astrocytes to form a reactive glial scar, while also influencing endothelial responses that alter the BBB permeability [48]. These interactions are essential for maintaining the CNS homeostasis and can significantly impact the extent and type of immune cell migration into the brain during ischemic events. The infiltration of peripheral immune cells into the CNS is a hallmark of the inflammatory response to CI [49,50]. The compromised BBB during AIS permits macrophages, neutrophils, and T cells to enter the brain [51]. Neutrophils, attracted by chemokines from ischemic tissue, arrive first [52]. While they remove cell debris through phagocytosis, they also release ROS and enzymes that can further damage tissue [53]. Macrophages follow, with roles that vary depending on their activation state. Generally, macrophages clear debris and secrete cytokines that can either amplify the local inflammatory response or help resolve it, depending on the milieu of signals they receive [54]. T cells, once recruited to the site, can exacerbate the inflammatory response or contribute to its resolution [55]. Specific subsets of T cells, such as regulatory T cells, can modulate the immune response to ischemia, reducing inflammation and potentially limiting damage [56]. The dual roles of these cells in both neuroprotection and neurotoxicity highlight the complexity of the inflammatory response in AIS. Effective therapies must therefore carefully balance these roles to minimize harm while maximizing the natural protective responses of the brain immune system. The mechanisms underlying neuroprotection and neurotoxicity involve complex signaling pathways. The release of neurotrophins by astrocytes can promote neuronal survival and plasticity, while the overactivation of microglial nicotinamide adenine dinucleotide phosphate (NADPH) oxidase (NOX) leads to excessive production of ROS, contributing to oxidative stress and cell death [57,58]. Targeting these specific pathways may help shift the balance towards neuroprotection, reducing the harmful effects of prolonged inflammation after AIS.

In the context of AIS, the inflammatory cascade within the CNS is often triggered and perpetuated by molecular signals indicative of cellular damage or pathogenic invasion. Two pivotal types of molecular players in this process are damage-associated molecular patterns (DAMPs) and pattern recognition receptors (PRRs) [59,60,61]. DAMPs are intracellular molecules released into the extracellular space following cellular damage or death, signaling tissue injury and initiating inflammation [62]. These molecules comprise various proteins, DNA, RNA, and metabolic byproducts that, despite their host origin, are potent activators of the immune response [62]. Their role is to alert the immune system to the need for intervention, either to control damage or initiate repair processes [63]. Prominent examples of DAMPs include proteins such as high mobility group box 1 (HMGB1) and heat shock proteins (HSPs), which can amplify the expression of pro-inflammatory genes, thus elevating the production of cytokines, chemokines, and mediators to sustain the inflammatory response [62]. Concurrently, PRRs, expressed on cells of the innate immune system, recognize both pathogen-associated patterns (PAMPs) and DAMPs, triggering immune and inflammatory responses [64]. Toll-like receptors (TLRs), a well-known class of PRRs, are crucial in this detection process [65]. TLRs are located on the cell surface or within intracellular compartments, recognizing a diverse array of molecules, from bacterial LPS and viral RNA to host-derived proteins like fibrinogen and HMGB1 [66]. This recognition activates signaling pathways such as the nuclear factor kappa-light-chain-enhancer of activated B cells (NF-κB) pathway, which orchestrates the transcription of genes involved in pro-inflammatory responses [67]. This activity is critical for mounting defenses against infection and for coordinating repair mechanisms after tissue damage. The role of TLRs in recognizing DAMPs becomes particularly significant following CI, as it contributes to the inflammatory response that, while initially protective, can become harmful if excessively prolonged or intense [68,69]. Recent advances in understanding and manipulating these inflammatory pathways offer new therapeutic opportunities. Clinical studies targeting specific PRRs, such as TLRs, have begun to show promise in moderating the inflammatory response in AIS [70]. Additionally, novel biomarkers derived from DAMPs are being explored to better predict AIS outcomes and tailor treatments to individual inflammatory profiles, potentially improving recovery rates and reducing long-term neurological deficits [71]. This dual nature of inflammation—both beneficial in clearing debris and harmful if uncontrolled—underscores the importance of understanding the balance mediated by DAMPs and PRRs. Such knowledge is crucial for developing therapies that can effectively modulate the innate immune response to improve outcomes in AIS and other CNS injuries.

### 2.2. Impact of External Factors: Role of Pathogens, Including Microorganisms and Their Components Like LPS, in Initiating Inflammatory Responses

Pathogens, including bacteria, viruses, fungi, and parasites, play significant roles in increasing the risk of AIS through mechanisms predominantly involving inflammation and vascular damage [72]. Particularly, bacterial infections have been extensively studied, showing a pronounced link to the AIS risk. Bacterial infections can exacerbate or trigger AIS by promoting atherosclerosis, increasing blood coagulability, and causing direct vascular injury [73]. For instance, *Chlamydia pneumoniae* is a well-documented bacterium found in atherosclerotic plaques, suggesting a direct involvement in the inflammatory processes that contribute to plaque formation and instability, leading to thrombosis [74]. Similarly, periodontal pathogens such as *Porphyromonas gingivalis* have been linked to a higher AIS risk due to their role in systemic inflammation [75]. These pathogens elevate levels of systemic inflammatory markers like C-reactive protein (CRP), which can lead to endothelial dysfunction and increase the vulnerability of blood vessels to atherosclerotic changes [76]. The bacteria produce endotoxins that activate various components of the immune system, promoting clot formation and vascular damage that are precursors to AIS [77]. Viral infections also contribute to the AIS risk, with herpes viruses, influenza, and COVID-19 being notable examples. Herpes simplex virus type 1 (HSV-1) and Varicella-zoster virus (VZV) are associated with cerebral vasculitis that can lead to vessel constriction and ischemic events [78,79]. Influenza, on the other hand, poses a transient yet significantly increased risk of AIS, particularly shortly after infection [80]. Similarly, COVID-19 has been linked to a heightened risk of AIS due to severe systemic inflammation and hypercoagulability [81]. The systemic inflammatory response, including elevated cytokine levels triggered by these viruses, enhances coagulation and can disrupt arterial plaques, leading to acute cerebrovascular events [82]. Fungal infections and parasitic infestations, though less commonly encountered, have also been associated with an increased risk of AIS, albeit through less frequently encountered mechanisms. Fungal pathogens such as *Cryptococcus neoformans* can lead to meningitis or CNS infections, particularly in immunocompromised individuals [83]. These infections can cause vasculitis or directly invade blood vessels, leading to AIS through vessel occlusion or rupture [84]. It has been found that parasites like *Toxoplasma gondii* and *Schistosoma* spp. may induce AIS through complex mechanisms involving systemic and cerebral inflammation, vascular damage, or by forming emboli that can obstruct cerebral blood flow [84,85].

LPS, key components of the outer membrane of Gram-negative bacteria, play a crucial role in systemic and neuroinflammation, which are significantly relevant to AIS pathogenesis [86]. LPS, consisting of a hydrophobic lipid A and a hydrophilic polysaccharide chain, is the main factor triggering the inflammatory cascade in response to infections with gram-negative bacteria [87]. LPS can activate the immune system by acting on immunologically competent cells, mainly monocytes, macrophages, and granulocytes, as well as platelets and endothelial cells [88]. It causes the release of inflammatory mediators: eicosanoids, cytokines, chemokines, adhesion molecules, ROS, platelet-activating factor (PAF), and NO [88]. LPS activates Toll-like receptor 4 (TLR4), initiating a cascade of immune responses [89]. When LPS binds to TLR4, it triggers the recruitment of adaptor proteins like myeloid differentiation primary response 88 (MyD88) and Toll/interleukin-1 receptor (TIR)-domain-containing adapter-inducing interferon-β (TRIF), leading to the activation of intracellular signaling pathways including NF-κB and interferon regulatory factors [89]. This signaling results in the transcription of genes that produce pro-inflammatory cytokines such as tumor necrosis factor-alpha (TNF-α), interleukin-1 beta (IL-1β), and interleukin-6 (IL-6) [90]. LPS can bind to plasma high-density lipoproteins (HDL) and lipopolysaccharide-binding protein (LBP) [91]. The formed complex has a high affinity for the cluster of differentiation 14 (CD 14) receptor present on leukocytes, monocytes, and macrophages, leading to their activation and inducing pathophysiological states such as inflammation or septic shock, which can be fatal. The consequences of infection result not only from the toxic activity of LPS but also from the activation of the host’s defense mechanisms [92].

Environmental and dietary factors also significantly influence the AIS risk through their effects on systemic inflammation [93,94]. Exposure to air pollution, specifically particulate matter (PM), is associated with increased systemic inflammation and a higher AIS incidence. Fine particulate matter, defined as particles that are 2.5 microns or less in diameter (PM2.5), can trigger inflammatory responses by entering the bloodstream via the lungs, exacerbating cardiovascular conditions and increasing the AIS risk, especially in urban settings [95]. Diet plays a crucial role in modulating inflammation. High-fat diets increase levels of inflammatory markers such as CRP and IL-6, which are linked to vascular risk and AIS [96]. Conversely, diets rich in fruits, vegetables, and omega-3 fatty acids, like the Mediterranean diet, have been shown to reduce inflammation and lower the AIS risk [97]. Specifically, omega-3 fatty acids and dietary fibers can decrease vascular inflammation and improve gut microbiota balance, contributing to reduced AIS susceptibility [98]. Thus, reducing exposure to air pollution and optimizing diets are vital for AIS prevention, emphasizing the need for lifestyle and environmental management in public health strategies.

### 2.3. Linking Neuroinflammation to AIS

AIS is characterized by a sudden reduction in blood flow to brain tissue, leading to an immediate deprivation of glucose and oxygen necessary for brain metabolism. [99]. As the global population ages and exposure to comorbidities increases, AIS poses a serious health threat and economic burden [100]. It is the second-leading cause of death and the third-leading cause of disability worldwide [1,101]. The average annual risk of second AIS after an initial stroke or transient ischemic attack (TIA) is 3–5% [102]. Patients with AIS or TIA are also at increased risk of subsequent myocardial infarction and vascular death [103,104].

The transition from systemic inflammation to neuroinflammation significantly impacts cerebral outcomes in AIS. This transition is facilitated by mechanisms that allow inflammatory mediators to influence brain pathology, primarily through BBB disruption and NVU actions [105,106]. Systemic cytokines, elevated during peripheral inflammation, can cross the BBB [107]. One mechanism involves increased BBB permeability during systemic inflammatory responses [107]. Cytokines like TNF-α and IL-1β weaken the tight junctions of the BBB, allowing inflammatory mediators into the brain’s extracellular space [107]. These cytokines also stimulate endothelial cells to express adhesion molecules, attracting peripheral immune cells [108]. These cells, once adhered, can transmigrate across the endothelium into the brain tissue, initiating a cascade of inflammatory processes that contribute to neuroinflammation [108]. The NVU, comprising endothelial cells, astrocytes, microglia, pericytes, and neurons, plays a pivotal role in maintaining cerebral homeostasis and responding to systemic inflammatory signals [109]. Under normal conditions, the NVU regulates cerebral blood flow, nutrient transport, and waste removal, and protects the neural tissue from toxic substances by maintaining the integrity of the BBB [110]. However, during systemic inflammation, the dysfunction of the NVU can lead to a breakdown in these regulatory processes, contributing to neuroinflammation [111]. The disruption is often exacerbated by the infiltration of activated immune cells and the local release of cytokines and chemokines within the brain, which can further damage the BBB and alter neuronal function [107]. This dysfunction can provoke a series of responses within the brain, leading to the activation of resident immune cells like microglia and astrocytes, which then contribute to the inflammatory milieu by releasing their own set of cytokines and chemokines [112]. These local responses not only aggravate neuroinflammation but also influence the progression and severity of neurological damage observed in AIS [113]. Thus, understanding the pathways involved in the transition of systemic inflammation into neuroinflammation and the role of the NVU in this process is crucial for developing therapeutic strategies aimed at mitigating the impact of inflammation on AIS outcomes.

Neuroinflammation plays a crucial role in the progression and outcomes of AIS, influencing both the acute and chronic phases. The sequence of events following ischemia—commonly referred to as the ischemic cascade—initiates with energy failure and progresses through excitotoxicity, oxidative stress, and culminates in inflammation, each step interlinked and exacerbating the next [114,115]. When cerebral blood flow is disrupted during ischemia, energy failure occurs due to a lack of oxygen and glucose, which are essential for adenosine 5′-triphosphate (ATP) production via aerobic metabolism [116]. This energy failure leads to the malfunction of ATP-dependent ion pumps in the neuronal cell membranes, resulting in an ionic imbalance [117]. Particularly, the failure to maintain membrane potential causes an excessive influx of calcium ions into cells. High intracellular calcium levels initiate a series of deleterious events, including the activation of enzymatic pathways that lead to cell damage and death [117]. This influx of calcium also triggers the release of glutamate, which is a major excitatory neurotransmitter in the brain [118]. Under normal conditions, glutamate facilitates neurotransmission, learning, and memory [119]. However, excessive glutamate during ischemia overactivates its receptors on neighboring neurons, leading to excitotoxicity [120]. This excitotoxicity further increases calcium influx, exacerbating cellular injury [120]. The excessive calcium and glutamate levels also enhance the production of ROS, leading to oxidative stress [121]. Oxidative stress compounds cellular injury by damaging proteins, lipids, and nucleic acids essential for cell survival [122]. The cumulative effect of these biochemical and molecular disruptions is a significant inflammatory response [122]. Inflammatory mediators released by damaged neurons and glial cells recruit peripheral immune cells to the site, intensifying the inflammatory response and often leading to further neuronal injury [123]. If the initial inflammation is not resolved after the acute phase of ischemia, it can transform into a chronic state, prolonging the neuroinflammatory response. [124]. Chronic neuroinflammation can have significant implications, both detrimental and potentially reparative, impacting recovery and long-term outcomes [125]. On the one hand, it can lead to ongoing secondary damage characterized by sustained release of cytokines and chemokines, persistent activation of microglia, and ongoing oxidative stress, all of which can contribute to further neuronal loss and functional impairment [126]. On the other hand, certain aspects of the inflammatory response post-stroke are crucial for initiating repair mechanisms, such as the clearance of debris by activated microglia and the secretion of growth factors that can help in the remodeling and regeneration of neural circuits [127]. However, the balance between damage and repair in chronic neuroinflammation is delicate and heavily influenced by the extent and location of the ischemia, the overall health of the neural environment, and timely therapeutic interventions [128]. Understanding the complex dynamics of neuroinflammation in CI, particularly the transition from acute to chronic inflammation, is essential for developing therapeutic strategies that can mitigate secondary damage while promoting recovery and repair. Effective interventions need to selectively target the detrimental aspects of inflammation while preserving or enhancing the beneficial processes essential for recovery and restoration of function in the post-stroke brain.

## 3. Metabolic Dysregulation and Inflammation: Adipokines and Obesity

### 3.1. Role of Inflammation in Obesity

Obesity is a condition characterized by an excessive accumulation of adipose tissue that exceeds the physiological adaptive capacities of the organism, representing a significant medical concern with profound implications for health [129]. It is a widespread health issue, impacting over 2.3 billion individuals globally, including both adults and children [130,131]. In clinical settings, body mass index (BMI) is predominantly used to assess weight issues, including obesity. A BMI range of 18.5–24.9 kg/m^2^ is considered normal. Values between 25.0–29.9 kg/m^2^ signify overweight, and a BMI of 30.0 kg/m^2^ or higher indicates obesity [132]. This classification helps to highlight the myriad of health risks associated with obesity, which extend beyond mere weight issues to more serious conditions such as diabetes, hypertension, and CVDs [133].

Unlike the localized and transient nature of acute inflammation caused by injury or infection, the inflammation associated with obesity is chronic and systemic [134]. This systemic inflammation is central to linking obesity to a range of metabolic and CVDs, including an increased risk of AIS [133]. In the clinical setting, systemic inflammation in obese individuals is typically assessed by measuring elevated levels of biomarkers in the blood, such as CRP and pro-inflammatory cytokines including TNF-α and IL-6 [135]. Elevated CRP levels, often found in obese individuals, are associated with an increased risk of developing chronic diseases such as type 2 diabetes, hypertension, and cardiovascular disorders [136]. TNF-α and IL-6 are not only pivotal in the inflammatory response but also directly exacerbate metabolic dysfunctions, leading to insulin resistance and the advancement of atherosclerosis, thereby linking obesity directly with increased cardiovascular risk [137].

In obesity, adipose tissue functions dynamically as an endocrine organ by secreting a complex array of molecules known as adipokines, which play critical roles in local and systemic inflammation as well as in metabolic regulation [138]. This secretion predominantly includes a mixture of pro-inflammatory cytokines, which contribute, to the chronic low-grade inflammation observed in obesity [139]. Significantly elevated levels of cytokines like IL-6 and TNF-α in the adipose tissue highlight an inflammatory milieu that further complicates insulin resistance, endothelial dysfunction, and other metabolic conditions [139].

Moreover, the rapid expansion of adipose tissue in obese individuals often leads to an inadequate blood supply, resulting in hypoxia [140]. Hypoxia, in turn, exacerbates the state of inflammation, promoting the production of even more inflammatory cytokines by activated macrophages within the tissue [141]. This chronic, low-grade inflammation is a key contributor to systemic effects, including altered glucose metabolism and a predisposition to atherosclerosis, significantly increasing the risk of CVDs such as AIS [142]. While hypoxia is generally considered detrimental due to its role in promoting inflammation, it is important to note the concept of hypoxic conditioning [143]. Hypoxic conditioning involves brief, controlled periods of hypoxia that can potentially induce protective mechanisms within tissues [143]. However, despite promising early research, large trials on hypoxic conditioning have not demonstrated significant clinical benefits [144].

Understanding the intricate mechanisms through which obesity leads to systemic inflammation, particularly through the dysregulation of adipokines, provides crucial insights into the pathophysiology of obesity-related diseases. It also highlights potential therapeutic targets to mitigate these effects, including interventions aimed at reducing inflammation and improving overall cardiovascular health outcomes. Addressing the inflammatory aspects of obesity involves not only managing weight but also understanding and modifying the underlying adipokine-mediated pathways that contribute to systemic inflammation and its broader health implications.

### 3.2. Adipokines in Detail

Adipokines include bioactive cytokines and hormone-like substances predominantly produced and secreted by adipose tissue [145]. They play critical roles in regulating metabolic processes, appetite, inflammation, and overall energy balance in the body [9]. The complex network formed by adipokines significantly influences the pathophysiology of obesity and associated metabolic disorders, highlighting a sophisticated interplay between metabolic health and inflammatory pathways [9].

Leptin, one of the most well-known adipokines, is notorious for its role in energy regulation and satiety [146]. However, leptin also possesses potent pro-inflammatory properties, which become particularly impactful in the context of obesity [147]. Elevated leptin levels, which are commonly observed in obese individuals, are correlated with increased adiposity and an enhanced inflammatory state [148]. Leptin enhances the production of inflammatory cytokines such as TNF-α and IL-6 by immune cells [149]. Furthermore, leptin contributes to the development of hypertension by increasing sympathetic nervous system activity and renal sodium reabsorption, and it promotes atherogenesis by encouraging the proliferation of vascular smooth muscle cells and the expression of inflammatory cytokines within arterial walls [150].

In stark contrast to leptin, adiponectin exhibits anti-inflammatory, anti-diabetic, and cardioprotective effects [151]. Unique among adipokines, adiponectin levels are inversely related to body fat percentage; they decrease as obesity increases [152]. This adipokine enhances insulin sensitivity, exerts protective vascular effects by inhibiting the transformation of macrophages into foam cells, and reduces the expression of adhesion molecules and pro-inflammatory cytokines in endothelial cells [153]. These properties help prevent the formation of atherosclerotic plaques and maintain vascular integrity, reducing the risk of CVDs and AIS [154].

**Figure 1:**
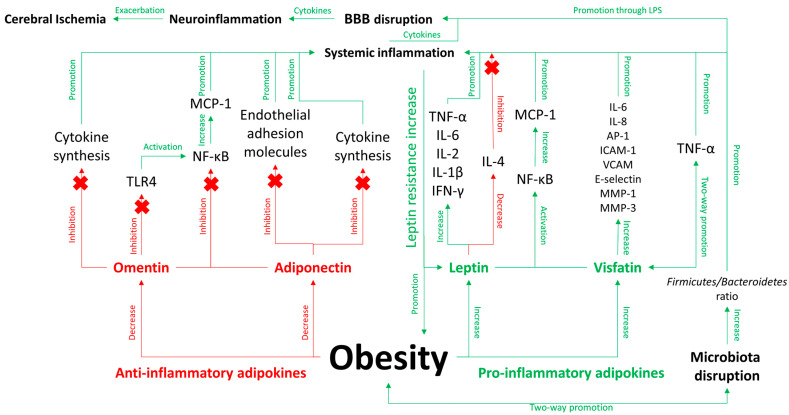
Complex interactions of inflammatory mediators associated with obesity are depicted, highlighting the influence of adipokine levels on anti-inflammatory and pro-inflammatory pathways. Adipokines such as omentin and adiponectin, which generally inhibit inflammation, are contrasted with leptin and visfatin, which promote inflammatory responses. Key components include the activation of nuclear factor kappa-light-chain enhancer of activated B cells (NF-κB) and the production of cytokines including tumor necrosis factor alpha (TNF-α), interleukin-6 (IL-6), and monocyte chemoattractant protein-1 (MCP-1). Additionally, disruptions in the *Firmicutes*/*Bacteroidetes* ratio, indicative of microbiota imbalance, further influence the inflammatory state by altering metabolic processes and exacerbating obesity-related inflammation. The figure emphasizes the progression from systemic inflammation to neuroinflammation. Disruption of the blood-brain barrier (BBB) by systemic inflammation and the cytokines that accompany inflammation may lead to neuroinflammation. This interaction highlights the role of gut microbiota disorders in amplifying systemic inflammation through mechanisms such as toll-like receptor 4 (TLR4) signaling and cytokine production, which is crucial for understanding the metabolic and vascular complications associated with obesity. AP-1—activator protein 1; ICAM-1—intercellular adhesion molecule 1; IFN-γ—interferon gamma; IL-1β—interleukin-1 beta; IL-2—interleukin 2; IL-4—interleukin 4; IL-8—interleukin 8; MMP-1—matrix metalloproteinase-1; MMP-3—matrix metalloproteinase-3; VCAM—vascular cell adhesion molecule 1.

The intricate balance between pro-inflammatory adipokines like leptin and resistin, and anti-inflammatory adipokines like adiponectin and omentin, underscores a pivotal aspect of obesity’s impact on health [155]. Understanding the roles and mechanisms of action of these adipokines not only illuminates the complex metabolic networks in obesity but also highlights potential targets for therapeutic intervention aimed at managing obesity and its numerous health complications. A deeper understanding of adipokine-mediated pathways is crucial for developing targeted strategies to reduce the inflammatory and metabolic risks associated with obesity and improve outcomes for individuals at risk of AIS and other serious cardiovascular events. The relationship between inflammation, obesity, and intestinal microbiota disruption is shown in Figure 1.

### 3.3. Direct and Indirect Pathways Linking Obesity to AIS

Obesity, as a body mass disorder, is more and more prevalent in both males and females worldwide [156,157,158,159,160,161]. One of its effects is chronic low-grade inflammation, which implicates it in the pathology of AIS [156]. Several pathways of adiposity-induced inflammation seem to be responsible for the prognosis and higher mortality in obese patients. Many of them begin in adipose tissue, the main fat-storing site in the human body. Hypertrophy and hyperplasia of adipocytes in obesity cause local stress, which directly leads to hypoxia, the process responsible for paracrine secretion of cytokines and chemokines. Among them, monocyte chemo-attractant protein 1 (MCP-1) attracts leukocytes to the tissue, stimulating further production of pro-inflammatory factors [156].

Another mechanism of obesity-induced inflammation is the activation of intracellular hypoxia inducible factor 1 alpha (HIF-1α), which in low oxygen conditions, characteristic of obesity, promotes the expression of genes involved in angiogenesis and apoptosis. Studies have shown that as the level of HIF-1α increases, the concentration of IL-6 also increases [156].

TLR4 is the next important factor exacerbating inflammation in AIS. Obesity causes changes in the microbiota, resulting in an increase in LPS, which in turn activates macrophages and microglia by TLR4, worsening inflammation [162]. Furthermore, TLR4 is responsible for the production of signal proteins that trigger obesity-associated changes, mainly c-Jun N-terminal kinase (cJNK) and NF-κB. Further, NF-κB plays a critical role in the formation of an inflammasome, consisting of a nucleotide-binding domain, a leucine-rich repeat pyrin domain containing 3 (NLRP3), an apoptosis-associated speck-like protein containing caspase recruitment domain (ASC), and procaspase-1. This structure allows for procaspase-1 activation, which in turn converts IL-1β and IL-18 from their pro-forms. This only deepens the inflammatory reaction in adipose tissue. Moreover, caspase-1 hydrolyzes gasdermin D (GSDMD). The product of this reaction, N-terminal cleavage of GSDMD, acts as a pore in cell membranes, which enables ions and fluids to migrate freely, resulting in swelling and pyroptosis, a type of cell death [156].

Studies have shown that a chronic high-fat diet may be related to higher levels of cytokines, including. IL-1, IL-6, and TNFα [156]. Furthermore, obesity induces gliosis, which consists of changes in the morphology and hyperproliferation of microglia and astrocytes, resident immune cells in the brain. Moreover, adipocity, associated with increased release of free fatty acids and cytokines, may cause a decline in BBB integration, which subsequently leads to further cerebral inflammation as cytokines and leukocytes can easily enter the CNS [156,162].

Certain mechanisms are not completely elucidated; however, it is generally considered that the exacerbation of AIS consequences in obesity is mainly related to their shared inflammatory pathways. This phenomenon may lead to excessive expression of pro-inflammatory factors and hyperactivation of immune cells, including microglia. These effects seem to further worsen AIS outcomes. Another possible mechanism for the role of obesity in AIS is reduced blood flow in cerebral arteries, which is most likely caused by the impairment of nitric oxide (NO)-mediated dilation [160]. Another detrimental impression of adiposity includes impaired efficacy of intravenous thrombolysis (IVT) by using recombinant tissue plasminogen activator (rtPA). However, the precise mechanism has not yet been ascertained. While some studies have denied the negative impact of obesity on IVT and endovascular thrombectomy (EVT), other studies have suggested that obesity might inhibit fibrinolysis, which in turn impedes clot dissolution, worsening the further prognosis and efficiency of the therapy [156,161,163].

In opposition to the above-mentioned evidence, some studies have described the so-called “obesity paradox” which may affect AIS patients. This paradox is based on the results implying that higher BMI is related to better outcomes of ischemic incidents in the brain. It should be emphasized that the studies presenting this phenomenon have important limitations. The correlation between BMI and AIS outcomes appears to be U-shaped, making it difficult to identify BMI values that are protective or detrimental to a patient’s outcome [160,161,164,165]. Nevertheless, more studies are needed to ascertain whether the obesity paradox in AIS is a real phenomenon.

Furthermore, there is also a question about the role of obesity in the pathology of AIS. Studies have shown that a BMI value above 20 increases the risk of AIS by around 5% per 1-unit rise in BMI [157,163]. Moreover, it has been proven that obesity increases the atrial fibrillation (AF) risk, which in turn is a potent cause of AIS [159]. However, there is also evidence that obesity may not have such a strong causal impact on the occurrence of AIS as on other cardiovascular diseases, which does not exclude its adverse impact on the development of the consequences of AIS [166].

### 3.4. Oxidative Stress in Metabolic Dysregulation—The Role of Reactive Oxygen Species in AIS Pathogenesis

Aging and chronic low-grade inflammation, often referred to as “inflamaging”, contribute to an increased production of ROS and a decline in antioxidant defense [167,168]. ROS arise from various sources, including pro-oxidant enzymes, radiation, lipid peroxidation, smoking, air pollutants, and glycoxidation [169]. The impact of ROS is extensive and varies depending on the source, type, location, concentration, and cellular targets, which determine whether the outcomes are physiological or pathological [169].

Under normal physiological conditions, ROS are essential for several biological functions, including lipid peroxidation, apoptosis, autophagy in damaged cells, gene regulation, glucose transport, and serotonin uptake [170]. They also influence the synthesis, release, and inactivation of endothelial-derived relaxing factor (EDRF), affecting vascular tone by either dilating or contracting blood vessels [171].

However, excessive ROS production can surpass the body’s antioxidant capacity, leading to a condition known as augmented oxidative stress [172]. This imbalance causes the oxidation of proteins—altering their structure and function, lipid peroxidation, nucleic acid damage, and depolymerization of hyaluronic acid, resulting in the accumulation of immunoglobulin G (IgG) [173]. Increased oxidative stress can also deactivate protease inhibitors, increasing the degradation of tissues. High levels of ROS can initiate molecular chain reactions that exacerbate damage to biomolecules, including the oxidation of polyunsaturated fatty acids in cell membranes [174].

At the molecular level, ROS are implicated in collagen degradation, disruptions in proteoglycan synthesis, enzyme inactivation, DNA strand breaks, chromosomal damage, and potential carcinogenesis [175]. Elevated ROS levels can disrupt mitochondrial oxidative phosphorylation, compromise the cytoskeleton, alter cellular antigenic properties, and disrupt intracellular calcium homeostasis [176]. Additionally, ROS can modulate the activity of various kinases and transcription factors, such as NF-κB, which triggers pro-inflammatory gene activation, promotes TNF-alpha-induced cell death, and sustains the activation of c-Jun N-terminal kinases (JNKs) by inhibiting mitogen-activated protein kinase (MAPK) phosphatases [177].

The body counters oxidative stress with enzymatic antioxidants like superoxide dismutases (SODs), glutathione peroxidases (GPXs), and catalase (CAT), as well as non-enzymatic endogenous and exogenous antioxidants, including vitamins A, C, E, glutathione, melatonin, and polyphenols [122]. These antioxidants are crucial for maintaining appropriate ROS levels and safeguarding against augmented oxidative damage, which is involved in aging and the development of age-related inflammatory diseases such as AIS [122].

The brain, particularly susceptible to oxidative damage due to its high peroxidable lipid content, low antioxidant activity, and significant oxygen consumption, experiences ROS-induced lipid peroxidation, protein denaturation, polynucleotide strand breaks, and base modifications [178]. These alterations significantly affect cell membrane permeability, ion transport, and BBB functions [179].

**Figure 2:**
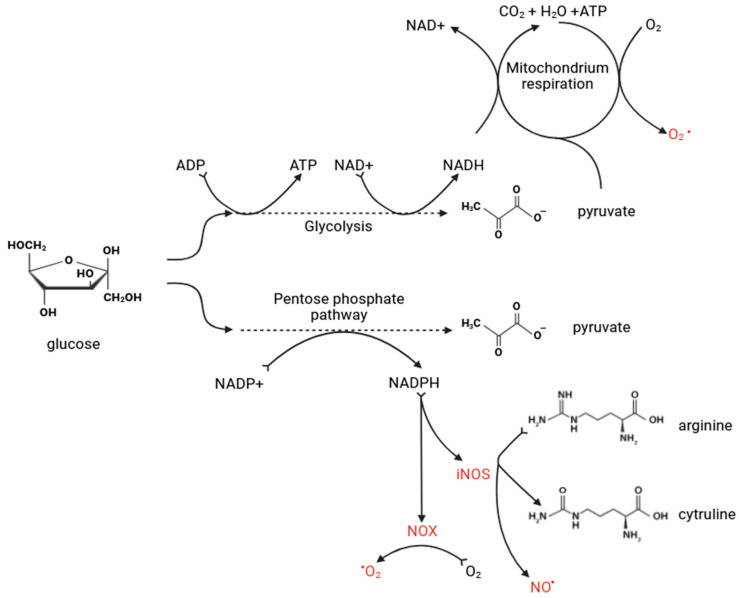
Major biochemical pathways leading to the production of reactive oxygen species (ROS) and reactive nitrogen species (RNS) within cells. It highlights glycolysis and the pentose phosphate pathway converting glucose to pyruvate and generating nicotinamide adenine dinucleotide phosphate (NADPH), which is utilized by NADPH oxidase (NOX) and inducible nitric oxide synthase (iNOS) to produce superoxide (O_2_^•^) and nitric oxide (NO^•^) radicals. These processes occur alongside mitochondrial respiration, which further contributes to ROS production through electron transfer mechanisms. Collectively, these pathways illustrate the critical role of metabolic activities in modulating oxidative stress and its impact on cellular health.

Figure 2 illustrates the primary biochemical pathways leading to the production of ROS and nitrogen reactive species (RNS), showcasing how glucose is metabolized through glycolysis and the pentose phosphate pathway, resulting in pyruvate that enters mitochondrial respiration. Additionally, it depicts the roles of inducible NO synthase (iNOS) and NOX in generating NO^•^ and superoxide radicals (O_2_^•−^), critical components of cellular oxidative stress.

In vitro studies have demonstrated that ROS can increase the permeability of brain endothelial cells, underscoring the critical impact of oxidative stress on the BBB integrity [180]. For instance, hydrogen peroxide (H_2_O_2_) heightens BBB paracellular permeability, accompanied by the rearrangement of Zonula occludens-1 (ZO-1) and increased production of occludin and actin proteins, ultimately leading to BBB disruption [181]. This disruption occurs in two stages: an initial breakdown due to endothelial dysfunction, followed by a later stage driven by inflammation, cell necrosis, and activation of matrix metalloproteinases (MMPs) [182].

## 4. Microbiota, LPS, and the Neuroinflammatory Cascade

### 4.1. Obesity-Induced Changes in the Gut Microbiota

The microbiota is such an integral part of the gastrointestinal tract (GIT) that it is sometimes referred to as an additional endocrine organ [183,184]. Collectively, it weighs around 1 kg, with the number of microbial genes exceeding human genes by a factor of 100 to 150 [184,185,186]. Statistically, the most dominant bacterial phyla in the human GIT, comprising more than 90%, include Bacteroidetes (Gram-negative, e.g., *Bacteroides* spp.), Firmicutes (Gram-positive, e.g., *Bacillus* spp., *Clostridioides* spp.), and Actinobacteria (Gram-positive, e.g., *Bifidobacterium* spp.); the dominant representative of the Archaea domain is *Methanobrevibacter smithii* [184,185,186,187,188]. Changes in the microbiota induced by obesity and a high-fat diet (HFD) reported in various studies are diverse, and some results are contradictory. However, the most common outcome seems to be a decreased abundance of Bacteroidetes with a concomitant increase in Firmicutes [162,187,188,189,190,191]. Evidence suggests that not only chronic changes in diet and body mass can alter microbiota composition. An HFD maintained for only three days has been found to shift the Firmicutes/Bacteroidetes ratio, with some changes observed within 24 h [187]. Conversely, a weight-reducing diet can modify the content of specific phyla, depending on the diet composition [187,192]. Moreover, physical activity shifts the Bacteroidetes/Firmicutes ratio in favor of the former [189]. The studies described strongly suggest that fat intake, overall diet, and body mass influence the microbiota in the GIT. Distinct microbiomal alterations, such as those found in obese patients compared to non-obese subjects, are often referred to as dysbiosis [183,185,187,191]. The main functional change observed in the gut microbiota of obese individuals appears to be an enhanced ability to extract energy from nutrients not digested by the human digestive system [185,186]. This mechanism has been shown to increase body mass in germ-free mice after transplanting microbiota into their GIT [186,187,193].

Many systemic alterations caused by changes in the microbiota are intricately tied to obesity, but it is unclear whether they are a cause or an effect of obesity. Research suggests a bidirectional relationship, where obesity promotes changes in the microbiota, which then further promote obesity [186,187,188]. Mechanisms of microbiota-induced systemic effects include fluctuations in intestinal permeability [184,187]. Gut microbes promote the proliferation of epithelial cells, greatly influencing absorptive function and barrier integrity in the intestine. Studies have confirmed that microorganisms can affect intercellular tight junctions, reducing permeability, and much evidence indicates the regulatory role of microbiota in this respect [187].

Short-chain fatty acids (SCFAs), produced by the gut microbiota, including acetate, propionate, and butyrate, significantly impact GIT functions [185,187]. Although the full mechanisms have not yet been elucidated, SCFAs serve as a primary energy source for epithelial cells in the colon, facilitating blood flow and hormone secretion, leading to higher stability of the intestinal mucosa [187]. Unfortunately, SCFAs also play a critical role in promoting obesity through multiple pathways. One involves increased triglyceride storage via the activation of carbohydrate-responsive element-binding protein (ChREBP) and sterol regulatory element-binding transcription factor-1 (SREBF1), and the suppression of fasting-induced adipocyte factor (FIAF) [185,191]. Another mechanism is specific to acetate, a precursor for acetyl-coenzyme A (acetyl CoA), a substrate for de novo lipogenesis. Excess acetate can cause obesity, steatosis, and further liver diseases [185]. The impact of SCFAs is complex because, alongside their negative effects, they also play roles in energy homeostasis, inflammation regulation, and increasing glucose tolerance by promoting the production of glucagon-like peptide 1 (GLP-1) [194].

Higher permeability caused by depleted gut microbiota primarily implicates lipopolysaccharides (LPS) and their pro-inflammatory effects [162,187].

### 4.2. LPS as a Bridge to Neuroinflammation

The first line of defense against microorganisms is non-specific immunity, involving cells such as macrophages, monocytes, and dendritic cells (DC) [195]. Recognition of microorganisms as invaders is possible due to interactions between structures that are part of PAMPs and PRRs [196]. The PAMP group includes surface proteins, lipids, sugars, and their complexes, such as glycoproteins and LPS [197]. TLRs, one of the best-studied PRR receptors, are involved in recognizing bacterial ligands and initiating the inflammatory process [87].

TLRs can recognize the pathogen and thus provide an almost immediate inflammatory response [198]. In the CNS, TLRs are primarily expressed on microglia and astrocytes, but they can also be found on oligodendrocytes, neurons, and Schwann cells [199]. Since TLR4 interacts with lipid molecules, lipid A, the part of LPS responsible for its toxicity, binds to this receptor [200]. The controlled accumulation of inflammatory factors such as prostaglandins, leukotrienes, TNF, and interleukins (e.g., IL-1, IL-6, IL-8, IL-10) has a beneficial effect on fighting microorganisms [3,201]. However, an excessive pro-inflammatory response due to overstimulation may lead to dangerous disease syndromes, including sepsis and septic shock [87]. The systemic release of these mediators not only targets invading bacteria but also causes widespread inflammation that can exacerbate vascular conditions like atherosclerosis or increase the risk of thrombosis, both of which significantly increase the AIS risk. The integrity of the BBB is crucial for maintaining CNS homeostasis, and systemic LPS exposure can severely compromise this barrier [202]. The permeability of the BBB increases due to the loosening of tight junctions between endothelial cells and the elevated expression of adhesion molecules that promote the adhesion and transmigration of leukocytes [203]. This disruption allows peripheral immune cells and inflammatory molecules to infiltrate the brain, where they can induce further inflammatory responses and contribute to the neuronal damage typical of AIS outcomes [17].

A properly functioning intestinal barrier prevents local and systemic consequences by blocking the translocation of bacterial products into the systemic circulation [204]. The intestinal barrier consists of multiple layers and serves as a physical and functional barrier to the transport of intestinal luminal contents into the systemic circulation [204]. While the epithelial cell layer and mucin constitute the physical barrier, intestinal alkaline phosphatase (IAP) produced by epithelial cells acts as a functional barrier by detoxifying bacterial endotoxin LPS and catalyzing the dephosphorylation of the active/toxic moiety of lipid A, thus preventing local inflammation and the translocation of active LPS into the systemic circulation [92].

Disruption of the intestinal barrier results in increased transport of LPS into the systemic circulation [204]. In the blood, LPS is carried either with LPS-binding protein (LBP) or with lipoproteins and interacts with surface receptors (e.g., TLR4) on immune cells, including microglia and astrocytes within the CNS, initiating an inflammatory response [204]. Upon reactivation, microglial cells produce pro-inflammatory cytokines, including TNF-α, IL-1β, IL-6, IL-12, IL-1α, complement component 1q (C1q), prostaglandin E2 (PGE2), and free radicals such as NO and ROS, to provide protection against pathogens [205].

Interestingly, in addition to pro-inflammatory factors, stimulated microglia also produce IL-10, a strong anti-inflammatory cytokine. While only small amounts of IL-10 are detectable in the early stages of neuroinflammation, its role in modulating the inflammatory response at this stage is crucial. The production of IL-10 by microglia increases over time and contributes to the resolution of inflammation [206].

Long-term activation of microglia increases oxidative and nitrosative stress and mitochondrial dysfunction, leading to neuronal death and neurodegeneration. Moreover, research by Liddelow et al. [207] showed that chronic pro-inflammatory stimuli originating from microglia, including TNF-α, IL-1α, and C1q, lead to the transformation of resting astrocytes into neurotoxic reactive astrocytes, responsible for killing neurons and differentiated oligodendrocytes.

Due to their immunogenic potential, microglia and astrocytes play key roles in nervous system inflammation. Their physiological activity ensures homeostasis and a precise response to pathogen invasion; however, uncontrolled and excessive reactivation has neurotoxic effects. Literature shows that nervous system inflammation is closely associated with chronic degenerative neurological diseases such as Alzheimer’s disease (AD) [208] and Parkinson’s disease (PD) [209]. Neuroinflammation also impacts amyotrophic lateral sclerosis, multiple sclerosis [210], and brain cancer [211]. Recently, the role of inflammation in the pathogenesis of acute-onset diseases such as AIS has been increasingly emphasized.

### 4.3. The Gut-Brain Axis in AIS

The intestinal microbiota influences the homeostasis of the entire body by producing specific metabolites that regulate the functioning of the gastrointestinal tract (GIT), nervous system, and immune system [212]. It is essential for the maturation of the immune system, regulating both innate and adaptive immune responses by influencing lymphoid tissue development and modulating IgA-producing B cells, T helper cells (Th17), and innate lymphoid cells [213].

The gut microbiota changes with the age of the host. The method of delivery (vaginal birth versus cesarean section) and the type of feeding (breastfeeding versus formula feeding) influence the initial colonization process of the newborn’s intestine [214]. Vaginal delivery exposes the infant to the mother’s vaginal and gut microbiota, while cesarean delivery limits this exposure, potentially affecting the newborn’s microbial diversity [215]. Although these initial differences tend to diminish over time, they may have implications for the child’s immune development and susceptibility to certain conditions [216]. The introduction of solid foods into the diet in children, followed by sexual maturation and hormonal changes, further shapes the composition of the intestinal microbiota, which reaches its highest diversity in adults [217]. Understanding these factors is clinically relevant, as early microbial colonization can influence long-term health outcomes, including metabolic and immune-related diseases.

The microbiota plays a key role in maintaining homeostasis between the brain and the digestive tract, forming a critical part of the intestinal barrier. The intestinal barrier also includes layers of intestinal epithelial cells, enterocytes, endothelial cells, and structures of the lymphatic, circulatory, nervous, and immune systems (GALT, gut-associated lymphatic tissue) [218]. A properly functioning intestinal barrier isolates microorganisms in the intestine and selectively permits the passage of various types of pathogens, protecting against inflammation [219]. Dysbiosis, triggered by an unhealthy diet, certain medications, chronic stress, autoimmune diseases, or impaired intestinal perfusion [220], increases intestinal barrier permeability. This leads to antigen penetration into the lumen, GALT stimulation, and inflammation. The gut-brain axis involves continuous, bidirectional signaling between the GIT microbiota and the nervous system, maintaining homeostasis [221].

Microorganisms significantly influence the formation and function of the intestinal barrier. Dysbiosis can disrupt this barrier, triggering nervous and immune system responses, leading to clinical symptoms. Metabolic products of intestinal bacteria, such as amino acids and neurotransmitter precursors, have beneficial effects on the host [222].

Neurotransmitters like dopamine and serotonin influence cortisol secretion, which increases intestinal permeability, activates immune cells, and alters intestinal microbiota composition. This can activate the brain-pituitary-adrenal axis [223].

Abnormalities in the intestinal microbiota can trigger a cascade of events, including increased intestinal permeability, disturbances in peristalsis, and intestinal secretion. This leads to immune system dysfunction and the activation of the vagus nerve, a key component of the gut-brain axis [218]. The vagus nerve, a major component of the parasympathetic nervous system, transmits gut-derived metabolites and hormones to the CNS, influencing brain function and inflammatory responses [224].

The gut-immuno-brain axis plays a crucial role in AIS pathogenesis by maintaining homeostasis through a complex communication network between the gut microbiota, immune system, and CNS. Disruptions in this axis can trigger inflammatory responses that increase AIS risk [225].

Microbial metabolites like SCFAs (butyrate, propionate, acetate), produced by gut bacteria fermenting dietary fibers, have anti-inflammatory properties and can cross the blood-brain barrier to modulate microglial activity, influencing neuroinflammation [226].

The GALT produces immune cells and mediators that can influence CNS inflammation through cytokine release. Dysbiosis can lead to the translocation of bacterial products like LPS into the bloodstream, triggering systemic inflammation and exacerbating brain injury during AIS [12].

Experiments on animal models and observations of patients have shown that microbiota disorders are specific to various diseases. However, it is still unclear whether these changes are primary or secondary to the disease [227]. Studies on animals deprived of intestinal microbiota indicate its key role in early brain development and adult neurogenesis. Research continues on the impact of the intestinal microbiota on the CNS and the mechanisms linking brain-intestinal microbiota and stress-related disorders, or CNS diseases [228].

The impact of AIS on GIT functioning has been known for decades [229]. For instance, dysphagia, a common clinical problem in AIS patients, is associated with a poor prognosis, an increased risk of pneumonia, malnutrition, and higher mortality. Due to the significant relationship between diet and intestinal microbiota [230,231], as well as diet and post-stroke recovery [232], microbiota may be a potential therapeutic target to protect brain function after ischemic brain damage. Oral food intake in dysphagia patients changes the gut microbiota composition in AIS survivors [233], though it is unclear whether these changes improve recovery. The intestinal microbiota can increase the risk of cardiovascular events, including AIS. Conversely, AIS can cause dysbiosis and disrupt the epithelial barrier, leading to systemic infections [234]. This bidirectional gut-brain relationship involving the brain, gut microbiota, and intestinal tissue may be key to AIS occurrence.

Peh et al. [230], based on the literature review and analysis of 14 clinical studies, hypothesized that patients after AIS have a reduced diversity of the intestinal microbiome. In cases of experimental AIS (conducted on animals), it has been shown that the severity of AIS correlates with the composition of the intestinal microbiome, which in turn, as mentioned earlier, depends on factors such as diet and medications taken, as well as age. While these findings suggest a potential relationship between microbiome composition and AIS severity, causality has not been definitively established. For example, consumption of foods rich in L-carnitine and choline is associated with AIS onset, potentially through the production of metabolites like trimethylamine N-oxide, which may influence stroke risk. Conversely, consumption of fiber has been associated with an improved prognosis after AIS, likely due to beneficial metabolites derived from the intestinal microbiota, primarily SCFAs. These metabolites have been shown to have anti-inflammatory effects and may help modulate the immune response post-stroke. However, the precise mechanisms through which these dietary components and their microbiota-derived metabolites influence stroke outcomes remain an active area of research. More longitudinal and interventional studies are needed to fully understand the causative relationships and the impact of gut microbiota on the immune system in the context of AIS.

Microbiota-derived metabolites like γ-aminobutyric acid, norepinephrine, dopamine, serotonin, tyramine, and tryptophan can directly affect brain cells or communicate indirectly via vagal afferent fibers. Depleting oral tryptophan, which converts to serotonin (5-HT), impairs long-term memory formation. The intestine is the main site of 5-HT production, dependent on gut microbiota presence and composition [222].

Germ-free animals have reduced peripheral 5-HT levels due to lower expression of tryptophan hydroxylase 1 (TPH1), the rate-limiting enzyme in tryptophan-to-5-HT conversion in enterochromaffin cells. An immature microglial phenotype is observed in germ-free mice, impairing the innate central immune system [235]. However, these disorders can be restored by recolonizing the gut microbiota, highlighting its role in CNS pathways and brain functionality.

Additionally, metabolites like lactic acid and propionic acid influence behavior and memory impairment. Propionic acid administration in rats results in abnormal behavior and stereotyped movements [236].

Trimethylamine is released by intestinal microflora metabolizing choline, lecithin, and L-carnitine, found in dairy products, eggs, fish, and meat [237]. In the liver, trimethylamine is converted to trimethylamine-N-oxide (TMAO) by the host’s flavin-containing monooxygenase enzyme. TMAO, a well-known microbiota-derived metabolite, leads to CVDs, including AIS. A systematic review and meta-analysis of 17 clinical trials involving 26,167 patients over 4.3 ± 1.5 years showed a positive correlation between TMAO levels and adverse cardiovascular and cerebrovascular effects [238].

Dysbiosis influences AIS pathogenesis by affecting immune cells. It can polarize T cells into pro-inflammatory states, leading to their migration to the ischemic brain area and exacerbating infarction damage [239].

**Figure 3:**
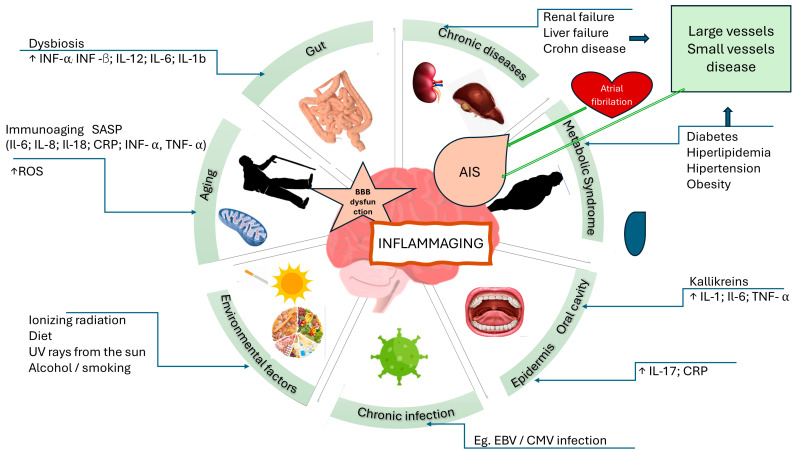
The diagram illustrates the various contributors to inflammaging and their interrelations with acute ischemic stroke (AIS). Factors such as dysbiosis, immunoaging, chronic diseases, metabolic syndrome, chronic infections, environmental influences, and aging contribute to systemic inflammation. Dysbiosis, marked by elevated inflammatory cytokines (interferon-alpha (INF-α), interferon-beta (INF-β), interleukin-12 (IL-12), interleukin-6 (IL-6), interleukin-1 beta (IL-1b)), and immunoaging, characterized by increased levels of senescence-associated secretory phenotype (SASP) factors (interleukin-6 (IL-6), interleukin-8 (IL-8), interleukin-18 (IL-18), C-reactive protein (CRP), interferon-alpha (INF-α), tumor necrosis factor-alpha (TNF-α)) and reactive oxygen species (ROS), play pivotal roles. Chronic diseases (e.g., renal failure, liver failure, Crohn’s disease) and metabolic syndrome conditions (e.g., diabetes, hyperlipidemia, hypertension, obesity) further exacerbate inflammation. These systemic inflammatory responses can lead to blood-brain barrier (BBB) dysfunction, promoting neuroinflammation and heightening the risk of AIS. The figure also highlights the role of atrial fibrillation and the interaction between environmental factors (e.g., diet, smoking, ultraviolet (UV) radiation) and chronic infections (e.g., Epstein-Barr virus (EBV), cytomegalovirus (CMV)) in the inflammatory process.

Current data strongly confirm the complex relationship between diet, intestinal microbiota, and their metabolites on the immune system and AIS development, course, extent, and recovery. Figure 3 presents the various contributors to inflammaging and their interrelations with acute ischemic stroke (AIS), illustrating the complex pathways leading to systemic and neuroinflammation.

## 5. Unraveling AIS Mechanisms: Etiology, Neuroinflammation, and Diagnostics

### 5.1. Influences on AIS Etiology—Beyond Traditional Risk Factors

AIS is a heterogeneous condition; different stroke subtypes have very different pathophysiologies and respond differently to treatment. Classic risk factors for AIS, including age, male sex, hypertension, AF, diabetes, hypercholesterolemia, smoking, and genetic predisposition, do not fully explain the pathogenesis of AIS; therefore, a detailed analysis of immunological phenomena accompanying the basic risk factors is also of increasing interest to researchers [240,241].

In clinical practice, only the probable cause of AIS can be determined, as the presence of a specific risk factor, such as AF, does not necessarily imply it led to the incident. The commonly used Trial of Org 10172 in Acute Stroke Treatment (TOAST) classification distinguishes the following etiological categories of AIS: LAA, CE, small artery occlusion (SAO), stroke of other determined cause (SOC), and stroke of undetermined cause (SUC) [242]. For each category, detailed clinical criteria are established, which suggest that a specific pathophysiological mechanism is most likely responsible for the occurrence of a cerebrovascular accident. Recent advances have linked molecular processes related to endothelial dysfunction, NO synthesis, BBB integrity, extracellular matrix maintenance and repair, and inflammation to these stroke categories [243].

Approximately 20% of AIS cases are attributed to LAA, which can precipitate stroke through two primary mechanisms: hypoperfusion due to hemodynamic stenosis or atherosclerotic embolism, where thrombus formation occurs following plaque rupture or ulceration in the vessel. Endothelial dysfunction is a recognized precursor to the pathogenesis of atherosclerosis [244]. Endothelial cells secrete various vasoactive substances that regulate coagulation, fibrinolysis, inflammation, and intercellular interactions. NO is crucial for endothelial function, enhancing tissue perfusion and reducing vascular resistance through cyclic guanosine monophosphate (cGMP)-mediated smooth muscle relaxation. Additionally, NO has cardioprotective, antihypertrophic, and antiproliferative properties, and it inhibits platelet adhesion and aggregation. The primary driver of endothelial dysfunction is often a reduction in NO production or availability, coupled with an increase in ROS [245,246,247].

The endothelial barrier, permeable to lipoproteins smaller than 70 nm, allows their passage from the circulation into the arterial intima. The interaction of apolipoprotein B (apoB)-containing lipoproteins with intracellular proteoglycans traps these lipoproteins subendothelially, leading to higher concentrations. These retained lipoproteins are susceptible to modification by oxidative agents, proteases, and lipases, producing oxidized phospholipids (oxPLs) and other lipid mediators. These mediators are pivotal in inducing leukocyte recruitment and activation, thereby exacerbating intimal inflammation [248,249]. OxPL, particularly derived from oxidized low-density lipoprotein (oxLDL), activates endothelial cells, influencing endothelial barrier functions, and is taken up by the CD14 receptor on dendritic cells, triggering inflammasome-dependent hyperactivation of phagocytes [250].

Monocytes and T lymphocytes adhere to endothelial cells due to the increased expression of adhesion molecules like vascular cell adhesion molecule 1 (VCAM-1) [251]. Chemotactic cytokines, primarily MCP-1 and its receptor—C-C chemokine receptor type 2 (CCR2), guide monocytes to the arterial intima, where they transform into macrophages. Then, these macrophages internalize modified lipoproteins to become foam cells, which produce ROS and MMPs, pro-inflammatory cytokines, and coagulation initiators [251]. Activation of the MAPK pathway increases the secretion of plasminogen activator inhibitor 1 (PAI-1), which impedes the conversion of plasminogen to plasmin, reducing fibrinolytic activity and promoting thrombus formation. Elevated PAI-1 gene expression in adipocytes is influenced by free fatty acids and pro-inflammatory cytokines [251].

Atherosclerotic plaques exhibit increased expression of components like the NLRP3 inflammasome, which is activated by microbial components such as LPS from oral and intestinal pathogens, as well as endogenous factors like ATP and palmitate [252,253]. Activation of the NLRP3 inflammasome in endothelial cells enhances endothelial permeability. These mechanisms underscore the role of NLRP3 inflammasome activation in human atherosclerosis, which is pivotal for intimal inflammation driven by cholesterol accumulation and mitigated by HDL-mediated cholesterol efflux [254].

Inflammation in atherosclerosis is not only initiatory but also undergoes an active resolution phase critical for maintaining immune homeostasis [255]. Eicosanoids, metabolic products of unsaturated fatty acids, regulate inflammation. Pro-inflammatory leukotrienes initiate and sustain inflammation, while specialized pro-resolving mediators (SPMs), including lipoxins and resolvins, promote resolution and restore homeostasis [256]. Changes in eicosanoid classes significantly affect the inflammatory response duration, with increased 5-lipoxygenase (5-LOX) expression correlating with plaque instability in carotid arteries and elevated mRNA levels following recent ischemic events [257].

Vascular smooth muscle cells (VSMCs) are central to atherosclerosis pathogenesis, with platelet derived growth factor (PDGF) playing a significant role in inducing VSMC phenotypic shifts from contractile to proliferative states. PDGF-driven changes in insulin receptor substrate (IRS) signaling in VSMCs, affected by phosphorylation levels, have implications for both nondiabetic and diabetic states [257,258]. Furthermore, recurrent acute infections or periodic reactivation of chronic infections can exacerbate atherosclerotic vascular disease, intensify hypertension development, and increase AIS risk by advancing atherosclerotic plaque progression.

AF contributes to 22–30% of AIS cases and is increasingly acknowledged for its inflammatory pathogenesis as a major factor in AIS development [259]. Elevated CRP levels in patients with AF are linked to both the incidence of AF and its recurrence post-ablation or cardioversion. AF induces a prothrombotic state through complex interactions known as Virchow’s triad: hypercoagulability, structural abnormalities, and blood stasis [260]. This state is exacerbated by the activation of the coagulation cascade, heightened platelet reactivity, and compromised fibrinolysis, further intensified by underlying comorbidities. Prothrombotic biomarkers such as platelet factor 4, von Willebrand factor, fibrinogen, β-thromboglobulin, and D-dimer are pivotal in this process. Structural changes like atrial fibrosis and endothelial dysfunction foster AF development, promoting atrial remodeling and setting the stage for clot formation and embolization. These factors are compounded by diminished blood flow due to chamber dilation and loss of atrial contraction. Although successful cardioversion significantly reduces levels of certain prothrombotic biomarkers like D-dimer, it does not affect fibrinogen levels. The relationship between AF duration and thrombogenesis remains contentious; recent studies have suggested that even brief episodes of AF can significantly enhance thrombogenesis, lasting up to 30 days without further AF recurrence [261,262].

Most studies on AF have examined peripheral blood samples; however, thrombus formation predominantly occurs in the left atrium (LA), where levels of coagulation, fibrinolysis, and fibrosis-related biomarkers are elevated compared to peripheral samples from AF patients. Thus, assessing prothrombotic biomarkers in LA blood samples might provide a more sensitive measure of the hypercoagulable state in AF [263].

The role of platelet activation in AF’s prothrombotic state is debated. While vascular damage in AF initiates primary hemostasis with prothrombotic markers driving vasoconstriction and platelet adhesion, activation, and aggregation [264], some studies, like those by Nagao et al. [265], have shown no differences in platelet activity biomarkers between AF patients and controls in the context of CE. These findings have been supported by the data from the Stroke Prevention in Atrial Fibrillation III study, which indicate that elevated β-thromboglobulin levels are not linked to thromboembolism [266]. Such studies underscore that peripheral blood samples may not fully reflect the actual platelet activation in the LA, yet this hypothesis might be validated by the ineffectiveness of acetylsalicylic acid (ASA) in preventing cardioembolic events in AF patients. Changes in LA morphology with endothelial denudation serve as a basis for microscopic thrombi that herald thromboembolic events [267].

AF, affecting 14% of the adult population, predominantly the elderly, accounts for over 79% of all cardiogenic AIS cases [268,269]. AIS related to AF typically results in poorer outcomes and higher costs than AIS from other etiologies [270]. Up to 40% of AIS cases remain cryptogenic, with their cause unidentified after routine evaluation [271,272]. Given the often paroxysmal nature of AF, conventional diagnostics like a single 12-lead electrocardiography (ECG) or 24-h Holter monitoring may fail to detect a significant proportion of patients with paroxysmal AF (PAF) [273]. Advances in extended ambulatory cardiac monitoring have improved the detection of PAF, enhancing the identification of patients who could benefit from anticoagulation to prevent secondary AIS, although practical limitations often hinder such diagnostics [274]. Current research aims to pinpoint biomarkers that can predict AF in AIS patients, with some inflammatory markers like CRP and IL-6 linked to recurrent AF [275].

CSVD typically arises from common risk factors such as hypertension, diabetes, and hypercholesterolemia [276]. However, in rare instances, it may have a genetic origin, such as in amyloid angiopathy, or an autoimmune basis, as seen in collagenosis. CSVD primarily impacts the small perforating vessels in the brain’s white matter and subcortical structures, as well as the microcirculation vessels [277]. Vascular pathology in CSVD may remain clinically silent or manifest as cognitive impairment, dementia, depression, gait disturbances, symptoms of lacunar stroke, or cerebral hemorrhage [243].

To date, the challenge in CSVD research has been the limited number of sufficiently powered and high-quality randomized clinical trials, with inconsistencies in trial methodologies complicating the interpretation of the results. Under the guidance of the International Society for Vascular Behavioral and Cognitive Disorders, the Framework for Clinical Research in Cerebrovascular Small Vessel Diseases (FINESSE) was established [278]. Additionally, the STandards for ReportIng Vascular changes on nEuroimaging (STRIVE) and subsequent STRIVE-2 criteria have been pivotal in defining the radiological features of CSVD, including updated definitions for imaging features such as recent small subcortical infarct, lacune of presumed vascular origin, white matter hyperintensity, perivascular space, cerebral microbleed, cortical superficial siderosis, brain atrophy, and the summary CSVD score [279,280].

Atherosclerotic changes and fibrillar necrosis in arterioles lead to arterial hardening and impaired contractility, resulting in increased vascular stiffness. This change is reflected in the pulsatility index (PI) assessed by transcranial Doppler ultrasound (TCD) in the internal carotid artery (ICA) and/or middle cerebral artery (MCA) [281,282]. Studies have shown that an elevated MCA PI is associated with severe white matter lesions and their progression in patients with lacunar infarction. It is also linked to cognitive impairment and infarct volume [283,284].

Endothelial cells in cerebral microvessels exhibit a specialized, non-fenestrated phenotype with extensive tight junctions and limited pinocytic vesicular transport. These cells are integral components of the NVU and the BBB, crucial for maintaining a stable environment and controlling cerebral homeostasis [285].

Table 1 provides an overview of various agents and their roles in acute ischemic stroke (AIS), microbiota dysfunction, and metabolic disorders.

### 5.2. Advancements in AIS Diagnostics

In recent decades, AIS diagnosis and patient prognosis have significantly improved due to advancements in imaging techniques like computed tomography (CT) and magnetic resonance imaging (MRI). However, widely available CT imaging has low sensitivity within the first 12 h post-stroke [270]. While MRI provides superior effectiveness, its availability is limited in many hospitals, and transporting patients to equipped facilities is time-consuming [270].

Advanced MRI techniques have unveiled functional abnormalities in blood vessels, with ultra-high-field MRI at 7 Tesla enhancing the imaging of individual perforating arteries and the measurement of their flow and pulsation velocities. A notable MRI method at this resolution allows for assessing blood flow and pulsations in the basal ganglia and the centrum semiovale’s perforating arteries, enhancing our understanding of CSVD’s severity in patients with lacunar infarction [311,312].

Diffusion tensor imaging (DTI) and structural network analysis have underscored the role of network disruptions in CSVD pathogenesis, which influence symptoms ranging from cognitive impairment to gait disturbances [243].

Disruptions in the BBB can be quantified through imaging that highlights the extravasation of contrast agents, enhancing image contrast. Techniques such as dynamic contrast-enhanced imaging on MRI or CT scanners allow for detailed assessments of BBB permeability, using paramagnetic or iodinated contrast agents [313,314].

Imaging techniques are now pivotal for identifying and evaluating the extent of glial activation, which becomes crucial in assessing the efficacy of immunomodulatory therapies. The positron emission tomography (PET)-detectable 18-kDa translocation protein (TSPO) serves as a primary marker for imaging activated microglia and macrophages, reflecting changes in mitochondrial functions essential for neurosteroid synthesis and other cellular processes [315,316]. TSPO expression patterns, which initially decrease post-stroke, peak in the subsequent weeks, and normalize over months, are indicative of ongoing microglial activation even in non-infarcted regions, extending up to a year after an AIS event [317,318]. However, the role of TSPO in neuroinflammation is considered controversial by some authors, as TSPO is expressed not only in microglia but also in astrocytes, endothelial cells, and vascular smooth muscle cells [319,320]. This broader expression profile complicates the interpretation of TSPO imaging data. Given these limitations, further efforts are focused on developing reactive glia-specific PET radioligands. For instance, [11C]CPPC, a high-affinity positron-emitting ligand specific for the macrophage colony-stimulating factor receptor 1 (CSF1R), has been developed. CSF1R expression is essentially limited to microglia in the brain, offering a more specific imaging target [321]. Despite the advancements, methodological issues persist in the use of MRI evaluations for neuroinflammation. These include challenges in accurately distinguishing between different types of glial cells and in quantifying the extent of neuroinflammation. Addressing these challenges is crucial for improving the reliability and specificity of imaging techniques used to study neuroinflammation in AIS.

Furthermore, PET imaging with cannabinoid type 2 (CB2) receptor ligands helps monitor therapeutic interventions aimed at modulating post-stroke inflammation. The energy demands of inflammatory cells, particularly in hypoperfused tissues where neurons and astrocytes succumb to ischemia, complicate the in vivo detection of metabolic deficits. The uptake of the PET tracer [18F]-2-fluoro-2-deoxy-D-glucose by these cells often masks the metabolic impairments in damaged areas [322].

Promising molecular targets in PET imaging for assessing post-stroke inflammation include α7 nicotinic acetylcholine and adenosine A1 receptors [323,324]. Lastly, the use of micron particles of iron oxide (MPIO) in MRI has gained popularity for molecular imaging, offering a biocompatible and non-immunoreactive method to identify endothelial activation in experimental AIS models [325].

Inflammatory activity can be assessed using various hematological markers derived from white blood cells (WBCs). The neutrophil-to-lymphocyte ratio (NLR) and platelet-to-lymphocyte ratio (PLR) have demonstrated increased predictive potential for CVD and related mortality [326]. Studies have also highlighted the predictive capacity of neutrophil counts for cerebral infarction, particularly in patients with vascular risk factors such as diabetes, smoking, and hyperlipidemia [327,328,329]. Furthermore, symptomatic carotid stenosis has been correlated with the presence of microemboli and increased neutrophil counts [330].

Inflammatory cells like leukocytes can induce proteolytic and oxidative damage to endothelial cells, enhancing hypercoagulability. The presence of low-density lipoprotein cholesterol (LDL-c), along with elevated plasma fibrinogen and serum IL-6 levels, is implicated in increased cardiovascular morbidity and mortality [291,292]. Neutrophil counts in peripheral blood are independently predictive of AIS severity upon admission, disability at discharge, and 30-day mortality [331].

The systemic immune-inflammation index (SII) and systemic inflammatory response index (SIRI) have emerged as new metrics, showing positive correlations with body inflammation and better predictive value for all-cause mortality and cardiovascular mortality compared to direct WBC counts [332,333]. Additionally, these indices have demonstrated associations with the risk of CVD and AIS severity, indicating their potential as biomarkers of prognosis [334,335].

In attempts to develop an immune risk profile (IRP), the OCTO/NONA project found that factors such as a CD4 to CD8 lymphocyte ratio less than 1, and the presence of cytomegalovirus (CMV) antibodies increased mortality risks, whereas a high CD4/CD8 ratio and a low percentage of CD8 + CD28—cells were indicative of “successful aging” [336]. Moreover, the CMV serostatus has been associated with an increased risk of all-cause mortality, particularly from cardiovascular causes, as shown in studies from the US and the UK [337]. However, in the BELFRAIL study, CMV serological status was not associated with an increased risk of mortality in the elderly cohort [338].

Local inflammatory markers and oxidative stress are critical for understanding endothelial dysfunction in AIS. Malondialdehyde (MDA) is widely used to assess lipid peroxidation, alongside measurements of antibodies against oxLDL (Ab oxLDL) and protein carbonyl groups, markers of protein damage by ROS [339]. Iron ions, which catalyze the formation of highly toxic hydroxyl radicals, also contribute to various types of DNA damage, including 8-hydroxy-2′-deoxyguanosine (8-OHdG), a marker for assessing oxidative DNA damage [339].

Lastly, epigenetic mechanisms such as DNA methylation have been found to play crucial roles in secondary brain damage following AIS. MicroRNAs, due to their stability in the blood and correlation with tissue levels, are promising as biomarkers. For instance, miR-137 has shown high diagnostic value for LAA stroke, surpassing traditional biomarkers in specificity [340]. The Tampere Vascular Study indicated elevated expression of miR-21, miR-34a, miR-146b-5p, and miR-210 in atherosclerotic arteries, underscoring their potential in diagnosing and understanding the pathogenesis of atherosclerosis [307].

## 6. Addressing AIS: Therapeutic Approaches and Future Directions

### 6.1. Current Therapeutic Strategies

Due to the diverse physiological conditions and multifactorial etiology of AIS, an effective treatment presents significant complexities and challenges. Currently, the most effective treatments include IVT using rt-PA and EVT [341]. These interventions aim to recanalize occluded vessels and restore blood flow to ischemic brain areas within a critical time window after ischemia onset. Despite these interventions, avoiding progressive neuronal degeneration and loss of function remains difficult. Therapeutic strategies are increasingly focusing on augmenting reperfusion therapy with treatments that modulate the pathological processes and immune responses involved in AIS recovery. To date, preclinical studies and clinical trials have predominantly explored avenues for neuroprotection and enhancement of post-stroke neurogenesis.

Since its approval in 1995, IVT with rt-PA has represented the gold standard for AIS treatment. Initial findings by the National Institute of Neurological Disorders and Stroke (NINDS) demonstrated consistent functional improvements across various AIS subtypes [342]. However, subsequent research has revealed that the etiopathogenesis and specific subtype of AIS significantly influence the efficacy of reperfusion treatments [343].

The success of thrombolysis largely depends on timely recanalization, which varies based on the thrombus size, composition, and origin. Cardioembolic clots, typically fibrin- and red blood cell-rich, are generally more amenable to the rt-PA treatment compared to the denser, platelet-rich clots formed from atherosclerotic plaques. While histological data are sparse, one in vitro study confirmed that fibrin- and red blood cell-rich clots are more susceptible to thrombolytic agents than their platelet-rich counterparts [344]. Additionally, the location of arterial occlusion and the inherent lytic susceptibility of the thrombus also play critical roles. For instance, proximal artery occlusions, like those in the distal internal carotid artery, are less responsive to thrombolytic therapy and are associated with poorer long-term outcomes [345]. Conversely, emboli originating from the left atrial are more likely to cause sudden, extensive vessel occlusion, leading to more severe consequences [346].

Early studies by Molina et al. [347] using transcranial Doppler (TCD) to assess MCA recanalization indicated that CE generally exhibit faster and more complete recanalization, correlating with better prognoses compared to other AIS subtypes [348]. Furthermore, patients with arterial stenosis of 50% or greater show less improvement at day seven post-IVT, although the AIS etiological subtype does not typically impact three-month outcomes [349]. Conversely, cardioembolic AIS patients tend to experience more hemorrhagic transformations and worse outcomes three months post-thrombolysis compared to those with LAA [350,351].

In studies focusing on the impact of diabetes and hyperglycemia at admission, results have shown that these conditions are associated with poorer prognoses in AIS patients, regardless of whether IVT is administered. Notably, higher glucose levels on admission are strong predictors of symptomatic intracranial hemorrhage post-intra-arterial thrombolysis, underscoring the critical influence of metabolic status on AIS outcomes [352].

Statins, a class of antihyperlipidemic compounds such as simvastatin, pravastatin, fluvastatin, and cerivastatin, inhibit 3-hydroxy-3-methylglutaryl-coenzyme A (HMG CoA) reductase, effectively lowering LDL and triglyceride levels while raising HDL in the blood. Recent research has consistently demonstrated that statin therapy significantly reduces the incidence of cerebrovascular events due to its lipid-lowering effects [353].

Beyond their role in cholesterol management, statins possess pleiotropic properties that set them apart from other lipid-lowering medications. These properties include anti-inflammatory, antioxidant, and immunomodulatory effects, which contribute to their ability to influence various essential processes in vascular biology [354]. Such effects are believed to regulate critical pathways that extend beyond cholesterol levels, impacting overall vascular health.

Further supporting their utility, the Heart Outcomes Prevention Evaluation-3 (HOPE-3) study revealed that combining antihypertensive therapy with statins significantly reduces the risk of a first AIS in individuals with moderate cardiovascular risk [355].

The development of therapies to mitigate or delay the progression of age-related neurodegenerative diseases remains a high priority due to the significant social, emotional, and economic burdens these conditions impose. Potential therapeutic strategies include anti-inflammatory medications and lifestyle interventions—such as dietary, exercise, and nutritional adjustments—that target age-related inflammatory immune phenotypes.

Endogenous neuroprotection is a primary focus in combating ischemic damage, enhancing the resilience of brain cells through the activation of intrinsic adaptive mechanisms [356]. HSPs, especially HSP70, have been identified as key players in numerous neuroprotective mechanisms in models of acute brain injury [357]. Research has elucidated HSP70-dependent endogenous neuroprotection strategies designed to halt mitochondrial dysfunction, activate apoptosis, desensitize estrogen receptors, and reduce ROS and RNS, thus preventing morphofunctional changes in brain cells during CI [358]. Developing modulators that enhance HSP70 expression is considered a crucial goal of contemporary pharmacology. Compounds such as tamoxifen, melatonin, glutamine, and pharmacological agents that augment HSP70 expression—like selenium compounds and glutaredoxin—are garnering significant attention for their neuroprotective potential [358].

The concept of microglial stimulation and its subsequent overreaction leading to secondary systemic inflammation has opened new avenues for treating neurodegenerative diseases by targeting systemic conditions or disrupting signaling pathways that mediate the CNS response to systemic inflammation. Both lifestyle modifications and pharmacological treatments show promise in slowing for halting neurodegeneration.

Recent studies have also highlighted the therapeutic potential of granulocyte colony-stimulating factor (G-CSF) in AIS [359]. Its neuroprotective and regenerative properties have been demonstrated in AIS models, and an early-phase study using human recombinant G-CSF to mobilize CD34+ cells showed promising results without systemic adverse effects [360].

Further research has indicated that reducing leukocyte and specifically neutrophil function can beneficially impact AIS pathology [361]. For instance, the use of anti-neutrophil monoclonal antibodies significantly reduces the production of free radicals and alleviates brain edema following ischemia-reperfusion injury [362].

Promising results have also emerged from studies on N-methyl-D-aspartate receptor (NMDAR) antagonists, compounds that reduce cytotoxicity, antioxidants, free radical scavengers, statins, antibiotics, and antagonists of pro-inflammatory cytokines [363]. However, it is important to note that, despite encouraging findings in animal models, many of these interventions have not succeeded in clinical trials [363]. These failures may stem from the biologically active substances having opposite effects depending on the disease phase.

Early-phase clinical trials evaluating agents like CD49d-specific antibodies and blockers of adhesion molecules like intercellular adhesion molecule 1 (ICAM-1), and macrophage 1 antigen (MAC-1) have not demonstrated effectiveness in clinical settings [364]. Conversely, astrocytes, the most abundant glial cells in the CNS, play critical roles post-stroke and represent promising therapeutic targets [365]. Research has shown that modulating the balance between pro-inflammatory and anti-inflammatory microglia can significantly affect ischemic outcomes [366]. For example, minocycline has shown potential neuroprotective effects in AIS, particularly in ischemic subgroups, though further randomized controlled trials are necessary to fully assess its efficacy and safety [367].

Despite many clinical trials yielding neutral or negative results, there are notable successes, such as the use of fingolimod, an anti-inflammatory treatment originally approved for multiple sclerosis [368]. Furthermore, an IL-1 receptor antagonist demonstrated promising results in a Phase II trial, reducing biological activity markers and improving clinical outcomes in patients with AIS [369].

Emerging therapies for CSVD are focusing on stabilizing endothelial function to prevent the long-term clinical and cognitive consequences associated with cerebral CSVD [243]. This approach highlights the ongoing efforts to address the complex etiologies of neurodegenerative conditions and underscores the potential of innovative treatments that may eventually translate into effective clinical practices.

### 6.2. Challenges and Opportunities in Therapy Delivery

The treatment of AIS presents multiple challenges that stem from the urgent nature of the condition, the diverse causes, and pathological changes it involves, and the long-term management required. One of the primary hurdles is the critical time window for effective intervention. Treatments such as IVT and EVT are most effective when administered shortly after AIS onset. Delays in recognizing AIS symptoms and accessing medical care can severely limit the effectiveness of these interventions.

The etiology of AIS varies widely among patients, influenced by factors like underlying CVDs, lifestyle, and genetic predispositions [370]. This variability necessitates personalized treatment approaches and complicates the standardization of care. Moreover, the physiological changes following AIS, such as excitotoxicity, inflammation, and oxidative stress, add layers of complexity to developing effective treatments [371]. The integrity of the BBB is often compromised, which not only impacts the pathophysiology of AIS but also restricts the delivery of therapeutic agents to the affected brain areas [314].

Developing drug delivery systems that can effectively target the ischemic penumbra and bypass or transiently open the BBB remains a significant challenge [372]. Even when medications reach the target site, their effects can vary based on the extent of damage and the individual’s response to treatment [372].

Long-term management of AIS includes dealing with the disabilities it often causes, which can vary widely among individuals. Rehabilitation efforts are tailored to individual needs and can be resource-intensive [373]. Psychological impacts such as depression or anxiety further complicate recovery and rehabilitation efforts [374]. Managing these effects is crucial for improving quality of life but is often an overlooked aspect of AIS recovery.

Prevention of recurrence is another critical area, requiring ongoing management of risk factors such as hypertension, diabetes, and lifestyle modifications. However, implementing and adhering to these changes can be challenging for many patients. The economic burden of AIS treatment, including the costs of acute care, long-term rehabilitation, and loss of productivity, as well as the emotional and physical strain on caregivers, places a significant load on individuals, families, and healthcare systems [375].

Addressing these challenges in AIS therapy requires a coordinated approach that includes advancements in medical treatments, healthcare system improvements, patient and public education, and policies that support comprehensive care strategies encompassing both physical and mental health aspects.

Regenerative medicine, focusing on repairing and replacing damaged cells through differentiation and proliferation, emerges as a promising alternative for addressing age-related neurodegenerative diseases. Stem cells, which are therapeutic beyond the acute phase of stroke, offer significant potential for extending interventional strategies across various patient profiles [376]. Rather than targeting the cause of AIS, stem cells aim to reverse or repair the ischemic damage. Due to their resistance to hypoxia, neural stem cells and precursor cells are particularly suited for surviving the critical hypoxic-ischemic phase and can differentiate into neurons, astrocytes, oligodendrocytes, and endothelial cells [377].

Among the most extensively studied stem cells are mesenchymal stem cells (MSCs), multipotent adult stem cells derived from bone marrow, adipose tissue, umbilical cord Wharton’s gel, amniotic fluid, and dental pulp [378]. Their ability to differentiate into various mesodermal lineages, coupled with their non-immunogenic nature, allowing for allogeneic use without the risk of transplant-induced teratoma, makes them particularly valuable [378]. The MSC secretome, rich in growth factors, cytokines, and anti-inflammatory agents, facilitates a range of therapeutic effects, from angiogenesis to anti-inflammatory actions [379]. The secretome’s paracrine signaling can activate pathways such as Akt/phosphatidylinositol 3′-kinase (PI3K), enhancing the local release of brain-derived neurotrophic factor (BDNF) and promoting cell growth, proliferation, and angiogenesis [380,381,382].

Endothelial progenitor cells (EPCs), circulating stem cells of endothelial origin, play a crucial role in vascular repair and remodeling following injury [383]. Mobilized by bone marrow in response to ischemia, EPCs help to restore damaged vessels through neovascularization [384]. They are characterized by specific endothelial markers and are instrumental in forming new vascular networks, mediated by factors like vascular endothelial growth factor (VEGF) and neurogenic locus notch homolog protein 1 (Notch1) signaling pathways. EPCs not only serve therapeutic roles but also function as diagnostic and prognostic biomarkers, with elevated levels observed in patients during the acute and subacute phases post-stroke [385,386].

Hematopoietic stem cells (HSCs), derived from bone marrow, peripheral blood, and umbilical cord blood, are capable of differentiating into all blood cell types [387]. Their differentiation, guided by factors like erythropoietin (EPO) and granulocyte colony stimulating factor (G-CSF), underscores their potential in treating blood-related pathologies associated with AIS [387,388].

In terms of administration, intravenous and intra-arterial routes are preferred, especially during the acute phase of stroke. Preclinical studies suggest that early intervention post-stroke yields better outcomes. While clinical evidence supporting stem cell efficacy in the hyperacute phase is limited, ongoing trials show promise for regenerative therapies within 72 h post-stroke onset. Notably, a multicenter phase II clinical trial (MASTERS) indicated the safety and tolerability of treatment with multipotent adult progenitor cells within the first 36 h of AIS onset, though significant improvements in neurological outcomes were not observed [376,389,390].

### 6.3. Future Research Avenues

Although preclinical studies have underscored the capability of medical imaging to assess neuroinflammation, these methods are not routinely utilized in clinical AIS diagnosis. Imaging of BBB permeability could be pivotal in identifying patients eligible for IVT or EVT outside of the conventional time windows, though standardizing image acquisition and analysis protocols remains a necessity.

The potential to image cellular or molecular markers of neuroinflammation could offer crucial diagnostic insights post-stroke. The development of biocompatible, biodegradable contrast agents that efficiently label human cells or bind specifically to human epitopes is critical for their successful clinical adoption. Targeting the nucleotide-binding oligomerization domain (NOD)-like receptor protein (NLRP3) inflammasome, which mediates inflammatory responses in AIS, including ischemia-reperfusion injury, presents a novel therapeutic avenue [301]. Numerous inhibitors of the NLRP3 inflammasome, including small molecules and various biological agents, have shown potential effectiveness [302].

Furthermore, microRNA-20b has been identified in animal models as a regulator of the NLRP3 protein, influencing ATP and ROS levels during CI [303]. Its modulation has been linked to reducing pro-inflammatory effects via the NLRP3 signaling pathway, presenting a significant area for therapeutic development.

In the realm of epigenetics, differential changes in long non-coding RNA and methylated DNA in the blood of AIS patients have shown promise as biomarkers for AIS risk, recurrence, and outcomes [391]. Epigenetic modulators are emerging as critical tools for tracking injury response and therapeutic efficacy. Post-stroke, several histone-modifying enzymes are dynamically regulated, influencing processes from cell death to neurodegeneration [392].

Regarding gut microbiota, advancements in high-throughput sequencing and metagenomics have elucidated the complex interplay between diet, microbiota, and metabolic diseases. Manipulating the gut microbiota through dietary changes, probiotics, and microbiota transplantation is becoming a viable strategy for treating inflammatory and metabolic conditions [393]. Probiotics have been shown to improve metabolic profiles and reduce inflammatory markers in various diseases, highlighting their potential in clinical applications [394].

Lastly, metabolic profiling has revealed distinct differences between LAA and CSVD groups, identifying specific metabolites that influence key metabolic pathways [395]. These findings underscore the potential of metabolomics in enhancing the understanding of AIS pathophysiology and guiding personalized treatment strategies.

## 7. Discussion

The present narrative review delves into the intricate interplay among metabolic dysregulation, gut microbiota alterations, and systemic inflammation, and their cumulative impact on AIS pathogenesis and outcomes. This discussion aims to synthesize the current understanding, highlight the ultimate goal of this research, and address the challenges, required knowledge and technology, significance, implications, strengths, weaknesses, and potential clinical applications.

The ultimate goal of this research is to elucidate the interconnected roles of metabolic dysregulation, gastrointestinal microbiome, and systemic inflammation in AIS. One of the primary challenges in this endeavor is the complexity of these interactions and their multifactorial influences on AIS risk and severity. Understanding the precise mechanisms by which these factors interplay remains a significant hurdle, necessitating advancements in research methodologies and technologies.

To overcome these challenges, advancements in neuroimaging, biomarker development, and computational modeling are crucial. These technologies will enable more precise mapping of the interactions between metabolic health, gut microbiota, and systemic inflammation, thereby improving diagnostic accuracy and facilitating targeted therapeutic interventions.

This research contributes to the growing body of literature that seeks to understand the multifactorial nature of AIS and the role of metabolic and inflammatory pathways. Understanding these pathways is significant as it opens new avenues for therapeutic interventions that can potentially reduce the burden of AIS. Integrating insights from metabolic, microbial, and inflammatory research is essential for developing holistic approaches to AIS management.

Our study builds on previous research by providing new insights into the specific mechanisms through which metabolic dysregulation and gut microbiota alterations influence neuroinflammation and AIS outcomes. By integrating findings from various studies, this review extends the current understanding and highlights potential areas for future investigation.

The findings from this study have broad implications for the development of targeted therapies aimed at mitigating the effects of metabolic dysregulation and systemic inflammation on AIS. Understanding these pathways can inform the design of novel therapeutic strategies that address the root causes of AIS rather than just managing symptoms. Furthermore, this research emphasizes the need for personalized medicine approaches that consider individual metabolic and microbial profiles in AIS treatment and prevention.

A key strength of this study is its comprehensive approach to understanding the multifaceted interactions among metabolic, microbial, and inflammatory factors. This integrative perspective provides a holistic understanding of AIS pathogenesis. However, limitations include the potential variability in patient microbiota and metabolic responses, which may influence the generalizability of the findings. Future studies should aim to address these variations through larger, more diverse cohorts and longitudinal designs.

The potential clinical applications of these findings include the development of personalized treatment strategies that target metabolic and inflammatory pathways to improve AIS outcomes. By identifying specific biomarkers and therapeutic targets, this research paves the way for interventions that can reduce the incidence and severity of AIS. Additionally, lifestyle and dietary modifications that promote a healthy gut microbiota and reduce systemic inflammation may serve as preventive measures against AIS.

In summary, this discussion underscores the importance of a multifaceted approach to understanding and managing AIS. By integrating insights from metabolic health, gut microbiota, and systemic inflammation, this research provides a comprehensive framework for future studies and clinical practices aimed at reducing the burden of AIS. Continued research in these areas is essential for developing effective preventive and therapeutic strategies that address the complex etiology of AIS.

## 8. Conclusions

This review article highlights the significant interplay between metabolic dysregulation, gut microbiota alterations, and systemic inflammation in the pathogenesis of AIS. Our findings demonstrate how adipokines, LPS, and ROS contribute to neuroinflammatory responses that exacerbate AIS outcomes. Understanding these interconnected pathways is crucial for identifying potential therapeutic targets to improve stroke prevention and treatment strategies.

By elucidating the roles of metabolic dysregulation and gut microbiota in AIS, we emphasize the importance of a multifaceted approach to stroke management. The review underscores how shifts in microbiota composition and systemic inflammation, often compounded by metabolic conditions like obesity, elevate the risk and severity of AIS. This comprehensive understanding offers new avenues for developing personalized interventions aimed at modulating these pathways to mitigate stroke impact and enhance recovery outcomes, ultimately improving the quality of life for those affected by AIS.

## Figures and Tables

**Table 1:** Key Agents Involved in Acute Ischemic Stroke, Microbiota Dysfunction, and Metabolic Disorders.

Name of Agent	Role of Agent in Acute Ischemic Stroke	Role of Agent in Microbiota Dysfunction	Role of Agent in Metabolic Disorders	References
Interleukin-1 alpha (IL-1α)	A strong activator of A1 astrocytes, which induce the death of neurons and oligodendrocytes.	-	-	[205,207]
Interleukin-1 beta (IL-1β)	Weakens tight junctions of the blood-brain barrier (BBB), causing it to unseal.	Studies showed that the microbiota can promote intestinal inflammation and pathology via NLRP3-mediated IL-1 signaling in mice.	Promotes inflammatory signals in surrounding cells, leading to adipose tissue inflammation.	[90,107,156,286]
Interleukin-2 (IL-2)	Secreted by activated microglia and TCD4+ cells in the acute phase of stroke, promoting inflammation.	Gut microbiota could modulate/mediate the efficacy of immunotherapies, which has been observed in cancer immunotherapies.	IL-2 is associated with insulin resistance as well as with several important inflammatory markers. In our obese cohort, two diverse clusters of IL-2 gene expression were identified, and in those with high expression, IL-2 gene expression correlated positively with fasting blood glucose (FBG) and glycated hemoglobin (HbA1c).	[155,287,288,289]
Interleukin-4 (IL-4)	Secreted by activated microglia and TCD4+ cells in the acute phase of stroke, promoting oligodendrogenesis.	In mice, extensive antibiotic treatment has caused elevation of IL-4 compared to those treated with probiotics.	Obese mice have lower levels of circulating IL-4; an 8-week IL-4 supplementation improves common phenotypes of obesity.	[155,289,290]
Interleukin-6 (IL-6)	Promotes proliferation of microglia and astrocytes, enhancing BBB permeability.	One of the most vital cytokines produced in response to inflammation.	Levels are elevated in obese patients and decrease after reduction in body mass.	[87,90,97,135,149,156,205,275,291,292]
Interleukin-8 (IL-8)	Mobilizes and activates neutrophils, causing their infiltration into the ischemic area, aggravating local inflammation, expanding ischemic lesions, and leading to severe morbidity and disability.	-	Diabetes mellitus is associated with higher levels of IL-8 compared to systemic healthy patients.	[87,155,293]
Interleukin-10 (IL-10)	A strong anti-inflammatory cytokine that plays a role in resolving inflammation.	IL-10 is emerging as a critical mediator of immunomodulation induced by microbiota, crucial for maintaining host-microbe homeostasis without triggering exaggerated detrimental inflammatory responses.	-	[87,206,294]
Interleukin-17 (IL-17)	-	Abrogation of the enteric IL-17RA signaling pathway led to commensal dysbiosis, increased serum GM-CSF concentration, and enhanced predisposition to neuroinflammation in mice.	Inhibits adipogenesis in mice.	[295,296]
Interleukin-18 (IL-18)	Highly expressed in atherosclerotic plaques, particularly in unstable plaques.	-	Elevated in subjects with metabolic syndrome and associated with its components, predicting cardiovascular events and mortality.	[156,297]
IFN-γ (Interferon gamma)	Secreted in the acute phase of stroke by Th1 lymphocytes.	Microbiota dysbiosis triggers the promotion of T-cell polarization into pro-inflammatory Th1 cells in the small intestine, migrating to the ischemic brain and exacerbating infarct damage.	-	[298,299]
TNF-α (Tumor Necrosis Factor alpha)	Secreted by activated microglia, forming neurotoxic astrocytes responsible for killing neural cells in neuroinflammatory settings.	A prominent pro-inflammatory agent that may contribute to septic shock due to extensive stimulation.	Exacerbates insulin resistance and metabolic disorders due to its pro-inflammatory properties.	[87,90,107,135,136,137,139,149,156,177,205,207]
MCP-1 (Monocyte Chemoattractant Protein-1)	Plays a critical role in attracting immune cells, especially macrophages, into adipose tissue, promoting the expression of pro-inflammatory proteins.	FMT from patients after bariatric surgery to recipients suffering from metabolic syndrome has been proven to decrease macrophage-attracting factor (MCP-1) in adipose tissue and plasma compared to FMT from donors with metabolic syndrome.	The circulating levels of MCP-1 and IL-8 in the serum were significantly (*p* < 0.05) higher in obese subjects (BMI > 30 kg/m^2^) compared with non-obese controls (BMI < 25 kg/m^2^).	[156,194,251,300]
NLRP3	Plays a pivotal role in intimal inflammation driven by cholesterol accumulation and in atherosclerotic plaques.	Activated by bacterial components, such as LPS.	-	[252,253,301,302,303]
Adiponectin	Prevents formation of foam cells.	Reduces production of pro-inflammatory cytokines.	Secreted by adipose tissue, levels negatively correlated with body mass, and has insulin-sensitizing properties.	[151,152,155]
Leptin	Elevated levels are found in sclerotic plaques, associated with angiogenesis and inflammation, contributing to atherosclerotic plaque burden and hypertension.	Dysregulated leptin levels influence gut microbiota composition, leading to an imbalance that exacerbates systemic inflammation and metabolic dysfunction	Increased leptin from adipose tissue acts in the hypothalamus to inhibit appetite and increase metabolic rate, reducing adiposity.	[146,147,194]
Omentin	-	-	Possesses anti-inflammatory properties similar to adiponectin, involving the activation of endothelial nitric oxide synthesis, regulating blood pressure, and influencing blood vessel relaxation.	[155]
Resistin	-	-	Increased levels indicate development of insulin resistance, diabetes, obesity, and cardiovascular disease; may be an early biomarker for metabolic disorders.	[155]
CRP (C-reactive Protein)	Chlamydia pneumoniae is involved in forming atherosclerotic plaques.	Porphyromonas gingivalis, a well-known bacterium, causes systemic inflammation, raising CRP levels, which have pro-coagulation and pro-thrombotic effects.	Elevated CRP levels are often found in obese individuals and are associated with an increased risk of developing chronic diseases such as type 2 diabetes, hypertension, and cardiovascular disorders.	[72,73,74,75,76,136]
Low-density lipoprotein cholesterol (LDL-c)	Its oxidized form plays a pivotal role in intimal inflammation and atherosclerotic plaque formation.	*Lactobacillus acidophilus* reduced the levels of malondialdehyde (MDA), oxidized low-density lipoprotein (ox-LDL), and TNF-α in the serum profile of treated mice.	LDL is one of the most prominent biomarkers of metabolic disorders.	[291,292,304]
MicroRNAs (miR-137, miR-21, miR-34a, miR-146b-5p, miR-210)	Levels of these miRNAs are elevated in atherosclerotic arteries and could be used as biomarkers in assessing vascular risk.	No circulating miRNAs are validated as biomarkers for routine use in clinical practice; lack of significant comparative studies between miRNAs and common disease biomarkers and high detection costs are the main limitations.	An association between alteration of miRNA expression profiles and progression of GI tract diseases enables the opportunity to explore new biomarkers in stool samples in the future.	[303,305,306,307]
Plasma fibrinogen	Its levels can be used as a predictor of carotid atherosclerotic plaque, ischemic stroke, and coronary heart disease.	Dysbiosis in microflora contributes to low-grade inflammation, causing coagulation disturbances.	A significant positive correlation was observed between fibrinogen concentration and HbA1c; negative correlation between fibrinogen concentration and HDL levels. Levels of fibrinogen are higher in obese patients with type 2 diabetes than in obese subjects without type 2 diabetes. Plasma fibrinogen levels correlate with fasting insulin levels and disease state advancement in non-insulin-dependent diabetics.	[291,292,308,309,310]
